# Acknowledgement to Reviewers of *JCM* in 2019

**DOI:** 10.3390/jcm9010287

**Published:** 2020-01-20

**Authors:** JCM Editorial Office

**Affiliations:** MDPI, St. Alban-Anlage 66, 4052 Basel, Switzerland; jcm@mdpi.com

Rigorous peer-review is the corner-stone of high-quality academic publishing. The editorial team greatly appreciates the reviewers who contributed their knowledge and expertise to the journal’s editorial process over the past 12 months. In 2019, a total of 2265 papers were published in the journal, with a median time to first decision of 18.95 days and a median time to publication of 36 days. The editors would like to express their sincere gratitude to the following reviewers for their cooperation and dedication in 2019:
A. A. DrososKen LiuA. John SimpsonKen MillsA.P. ShortlandKen RosenthalAaron Michael RulsehKen TakahashiAaron PiepmeierKenichi FukudaAbbie FrancisKenichi TakayamaAbdul MatinKenneth LimAbdullah Al MarufKenneth OoiAbdullah Al-ZaghalKenneth R. PhelpsAbed KhalailehKENSEI TAGUCHIAbheepsa MishraKensuke MatsushitaAbhinav AchrejaKensuke ShojiAbhinav VasudevanKent LundholmAbhishek MangaonkarKenta MurotaniAd SnikKentaro OkunoAda CheungKentaro TojoAdam AdlerKentaro YoshiokaAdam E. GawedaKenya KusunoseAdam J. BiałasKeqiang YeAdam J. SybilskiKe-qin HuAdam KowalczykKeren LevanonAdam PickardKerollos Nashat WanisAdam RaikesKerstin GruendlerAdam ReichKerstin StinglAdam StrzelczykKeshav Raj PaudelAdam SzpechcińskiKęstutis MaslauskasAdam W BarbKęstutis RimaitisAdamanthia LiapikouKeun-Yeong JeongAdil El MidaouiKe-Vin ChangAditya BansalKevin EboigbodinAditya DandekarKevin EliasAdolfo Di FioreKevin FlahertyAdomas BunevičiusKevin LobdellAdriaan HolleboomKevin SinghAdrián CeccatoKfir LapidAdrian CovicKhashayar SakhaeeAdrian ManKhosrow RezvaniAdriana J Van BallegooijenKhurram J. KhanAdriana RamosKhurshid AhmadAdriana RusuKi Chang KeumAdriano PiattelliKia AsbergAdrie VerhoevenKi-Chul HwangAdwitia DeyKieran HalloranAdwitiya KarKiichi HirotaAfshan N. MalikKi-Jong RheeAgata ChmurzyńskaKim BlomAgata Maria RabuazzoKim KultimaAgata StanekKim LawsonAgata SzulcKim MaddenAgathe ChaigneKim OrchardAgbangla Nounagnon FrutueuxKim Van RooijAgne TaraseviciuteKimberly ClaudatAgnes BerendsenKinda IbrahimAgnieszka AdamekKinga Grzech-LeśniakAgnieszka BieńKinga PolanskaAgnieszka GerkowiczKiran K. UpadhyayAgnieszka GniadekKiran UpadhyayAgnieszka JaworowskaKirsten HöttingAgnieszka Kolasa-WołosiukKirsti Uusi-RasiAgnieszka KuchtaKirstie Canene-AdamsAgnieszka MichaelKirstie K. DanielsonAgnieszka MickiewiczKishore ChallagundlaAgnieszka PacKishu RanjanAgnieszka Paradowska-GoryckaKismet Hossain-IbrahimAgnieszka PodfigurnaKivovics MártonAgnieszka ŚmieszekKiyofumi TakabatakeAgnieszka ZmysłowskaKiyohiko SakataAhlke HeydemannKiyoshi MoriAhmed AbassKiyoshi TakagiAhmed AbuzaidKiyoto ShigaAhmed Al-ImamKjetil AskAhmed AlTurkiKlara KomiciAhmed BakillahKlaus KayserAhmed Ba-SsalamahKnut TaxbroAhmed IbrahimKodani EitaroAhmed J. AljaafKOEHL BerengereAhmed NassrKoen Van Der BogtAhmed ShokerKoichi NakanishiAhmed ZakyKoichiro YanoAhmet BaydurKoji FujitaAhsan AkramKoji MatsuoAileen HillKoji MitaAin A. NeuhausKoji NaruishiAisha DickersonKoji SawadaAJAY PALKoji UchiyamaAjoe John KattoorKommidi HarikrishnaAke NilssonKondababu KurakulaAkella ChendrasekharKonstantin NeumannAkifumi HagiwaraKonstantin TsoyiAkihiko HiyamaKonstantinos C FragkosAkihiro OhmotoKonstantinos FourtounasAkiko MaedaKonstantinos FragkosAkiko ShimadaKonstantinos N. PaterakisAkio MitaniKonstantinos PapamichailAkira MonjiKonstantinos SapalidisAkira TsuchiyaKonstantinos T TsaousisAkira UmemuraKosaku MurakamiAkishi MomoseKosei NagataAkiyoshi KinoshitaKouichiro KawanoAkiyoshi TakamiKoyin ChangAkshay BarejaKrishan YadavAkshay BatraKrishna Kumar VeeravalliAkylbek SydykovKrishna M. SundarAla AltaieKrista J. SiefriedAlain BerthozKristen BubbAlain J PonceletKristen M. MeiburgerAlain Van GoolKristen RileyAlain VeilleuxKrister HåkanssonAlan FeingoldKristian M. HargadonAlan FraserKristien J. LedeganckAlan G. GoodmanKristiina RönöAlan HarperKristin HolvikAlan M. HanashKristin SellersAlan PALMERKristina C DuffinAlan ValapertiKristina G HultenAlan WuKristine M. KrajnakAlastair M BuchanKrisztina CsabafiAlbert A OkunadeKriti PuriAlbert PostmaKrystyna GolonkaAlbert Selva-O'CallaghanKrzysztof KrystaAlbert W. GirottiKuan Hao TsuiAlbert WölflerKuan-Fu ChenAlberto A. UribeKui XuAlberto AntonelliKumar VaibhavAlberto G. DistefanoKun-Ming ChenAlberto Marcos Heredia-RizoKuntal DeAlberto Martínez-CastelaoKun-yun YehAlberto OuroKuo-How HuangAlberto Rubén OsellaKuo-Sheng HsuAlberto SignoreKurita NaokiAlberto Soriano MaldonadoKurt AmmerAlberto TagliaficoKurt GrüngreiffAlbino CarrizzoKurt HecherAlcínia Z. SampaioKurt PiepenbrinkAlda MarquesKusumika MukherjeeAldo ClericoKwan Soo KoAldo RagazzoniKwang-Huei LinAlejandra Carolina LastraKwon Ok-SeonAlejandro BalsaKyle BurghardtAlejandro Lizaur-UtrillaKyle VanKoeveringAlejandro SanzKynan EngAleksandr KakinenKyongmee ChungAleksandra M. PavlovićKyoung Ah MinAleksandra Palatyńska-UlatowskaKyoung-Chan ParkAleksandra SzymczakKyoung-Jin OhAleksandra WilkKyoungmin ParkAleksandra ŻebrowskaKyu Chang WonAlena OpattováKyu Eun LeeAlena TichaKyu-Bok LeeAleš JerinKyung-San MinAlessandra CalogeroKyung-Yil LeeAlessandra CastaldiKyuseok KimAlessandra D'AzzoL. Maximilian BujaAlessandra DurazzoLadan GolestanehAlessandra FeracoLadislav VolicerAlessandra FerramoscaLaetitia DouAlessandra PaciniLaetitia M.O. De KortAlessandro AgostiniLakshya BajajAlessandro AlaimoLaleh MajlessiAlessandro BarboneLalit KauraniAlessandro BellettiLampros PapadimitriouAlessandro CaffòLan GaoAlessandro CastorinaLan YueAlessandro De TroiaLara EdbrookeAlessandro DelitalaLarry I. LipshultzAlessandro DidonnaLars Ailo BongoAlessandro FraldiLars P. KamolzAlessandro GuidottiLars TöngesAlessandro MalobertiLars WikAlessandro MarinelliLasse Dahl JensenAlessandro MatteLata JayaramAlessandro MeduriLaura AstolfiAlessandro PresentatoLaura BenagliaAlessandro RolfoLaura BuggioAlessandro SanduzziLaura C. ReigadaAlessandro VatrellaLaura ComermaAlessia CiarrocchiLaura E. L. PerkinsAlex CarllLaura E. MatareseAlex ChangLaura E. WatkinsAlex HeyseLaura FloreaAlex LeffLaura FouassierAlex Van 't HulLaura FreinaAlexander EgleLaura Giuseppina Di PasquaAlexander FanaroffLaura Katharina SieversAlexander FlanneryLaura Katharina SieversAlexander H. MaassLaura MasonAlexander HemprichLaura PetriniAlexander HicksLaura Pletsch-BorbaAlexander LinkLaura R. LachanceAlexander MachadoLaura SanchisAlexander MedvedevLaura StraussAlexander NeuweltLaure Sarda-MantelAlexander NgwubeLaureline BerthelotAlexander R. OpotowskyLauren AllanAlexander RothmanLauren C PeresAlexander TallnerLauren C. BlekkenhorstAlexandra AmaralLauren F. GoodmanAlexandra PapadopoulouLauren NicholasAlexandra-Maria MichaelidouLauren StewartAlexandre Brind'AmourLaurence JossetAlexandre JoostenLaurence KlotzAlexandre TrindadeLaurent GodinasAlexandros MakisLaurent GuyotAlexandru Florin RogobeteLaurent KintzeléAlexei WongLaurent M. RiouAlexey SarapultsevLaurent MetzingerAlexey SurovLaurent VergnesAlfonso Javier Ibáñez-VeraLaurie OzeliusAlfonso MastropietroLaurie TwellsAlfonso TorquatiLavinia Cosmina ArdeleanAlfredo PeralesLawrence H. LeAli BettaiebLawrence V. FultonAli Borzabadi-FarahaniLawrence H LashAli ErsenLazaros I. SakkasAli HaratiLazzeri GiacomoAli KhurramLea-Ann KirkhamAli Salehzadeh-YazdiLeah OwenAlice BrandliLeandro SastreAlice DomenichiniLee Ann Macmillan-CrowAlicia Guemez-GamboaLee Anne B. SherwinAlina DimaLee TanAlina GhinetLeesa M. PruntyAlina Simona RusuLei HuangAlina Simona RusuLei WanAlisa ArunamataLei WangAlisa JohnsonLei ZhangAlison BrattLeif Edward Ottesen KennairAlison KayeLeigh A. FrameAlison MunroLeili BehroozAlison Sally PoultonLena HaglinAlisson TelesLena IllertAlistair McEwanLeonard B. MaggiAllen L. HoLeonard BieloryAllison RosenzweigLeonardo PellicciariAllister GibbonsLeonie Marloes VogtAlmea MatanockLeopoldo FornerAlmudena Gómez-HernándezLeslie MeynAlois BonifacioLeslie NorinsAlphonse G. TaghianLester KobzikAlun C. JacksonLetizia De ChiaraAlus XiaoliLetizia FerroniAlvaro Joffre Uribe QuevedoLevi J. BeverlyAlvaro MesoracaLewis CarpenterAlvaro Rojas-PeñaLewis HsuAlyssa PanitchLewis J KaplanAmadeus DobrescuLianbo LiAmal O. AmerLi-Ang LeeAmanda BrignellLiang-Jen WangAmanda Fernández RodríguezLiang-jun YanAmanda J. NicollLídia CedóAmanda M. NelsonLidia SantarpiaAmanda TownsendLihua DongAmandeep AroraLikwang ChenAmany TawfikLili O. HorváthAmar PatelLilian Calderon-GarciduenasAmaricai ElenaLilian I. PlotkinAmaya Garcia CostasLiliana SachelarieAmber Parry-StrongLili-Naz HazratiAmedeo AmedeiLilja B. SolnesAmedeo LonardoLin ChenAmelia CarrLin ChengAmelia DrakeLin LiAmelia LicariLin ShihpinAmer M. ZeidanLina A. BegdacheAmerigo GiudiceLinda BrunotteAmgad HannaLinda HaddadAmin El-HeliebiLinda K. KayeAmin R. JaverLinda MaerzAmir R. KachooeiLindsay BodellAmira GuirguisLindsey CrawfordAmirali SalmasiLingxiao LiAmirhessam TahmassebiLinus OlsonAmit AkirovLionel RostaingAmit FrenkelLisa Harrison-BernardAmit KishoreLisa M. ReiderAmit KumarLisa MellhammarAmit Kumar SahaLisa PastoreAmit MistriLisal FolsomAmit SharmaLisandro Damian ColantonioAmith ShettyLise KvebergAmitkumar MehtaLiselotte MettlerAMMAR.B AltemimiLishuang ShenAmna UmerLi-Tung HuangAmosy E M’KomaLiujiang SongAmosy M'KomaLivia D'AngeloAmrita SarkarLivia De Lucca CamargoAmritha BhatLivio LuongoAmritlal MandalLiwei LangAmutha RamadasLixin LiAmy E. SobotaLiya WangAmy L. LovellLlanos CasanovaAmy LehrnerLluís PuigAmy MacNeillLobo LaraAmy O'SheaLois BalmerAna AdanLola Jade PalmieriAna BanitoLola RahibAna BujánLong ShenAna CandiotaLoredana BergandiAna I. Lopez NavasLoredana PucaAna Margarida UrbanoLorella BattiniAna MartinezLorena AiraghiAna MessiasLorenz Kadletz-WankeAna Rita CarlosLorenz KuesselAna SanchoLorenzo CavagnaAnabela BarcelosLorenzo DesideriAnais LacasseLorenzo DragoAna-Maria FloreaLorenzo FaggioniAnandharajan RathinasabapathyLorenzo SpaggiariAnannya BhattacharyaLorenzo TonettiAnanta PaineLoreta KuzmieneAnastasia MitseaLori BanksAnastasia ProdromidouLori E. LowesAnastasios KarayiannakisLothar LilgeAnastasios KoulaouzidisLouis PuybassetAnat GalorLouise BroughAnca DobreanLouise HullAnca E. MuşetescuLubica DudakovaAnca FranziniLuca AgnifiliAnca GafencuLuca AmpolliniAnca Oana DoceaLuca CasartelliAndaleb KholmukhamedovLuca CoppetaAnderly C. ChüehLuca De StavolaAnders GottsaterLuca De ToniAnders HenningsenLuca D'OnofrioAnders M. GreveLuca FerraroAnders Mark-ChristensenLuca FiorilloAnders R. ClausenLuca GiannellaAndrás BikovLuca LiberaleAndras FrankoLuca MadaroAndre IovaneLuca MorandiAndre NybergLuca MorelliAndré StruglicsLuca MunaronAndrea CaputoLuca PagliardiniAndrea ClementsLuca QuartuccioAndrea CochisLuca RevelliAndrea CordaLuca RomeoAndrea De MariaLuca TestarelliAndrea FurlanLucas A. SalasAndrea GragnanoLucia AnsaniAndrea IsidoriLucia GiombiniAndrea L. GropmanLucia Lucia MonacisAndrea LaniaLucía MéndezAndrea LeonardiLucia MolettaAndrea MartinuzziLucia MorbidelliAndrea MilenkovicLucia PacificoAndrea MohrLucia PasquiniAndrea PilloniLucia Pirisi-CreekAndrea SambriLuciano PacificiAndrea ScribanteLucie H ClappAndrea SurányiLucile GreggAndrea TicinesiLucio MangoAndrea TinelliLucy MarzbanAndrea TinnirelloLudovico AbenavoliAndrea VianelloLuigi BoccutoAndrea WeghoferLuigi BonavinaAndréanne LupienLuigi CodecasaAndreas BrodehlLuigi Taranto MontemurroAndreas F HottingerLuigi VetrugnoAndreas HeckerLuis E. Bello-EspinosaAndreas JunkerLuís F. MendesAndreas KalogeropoulosLuis G MedinaAndreas KammerlanderLuis MoitaAndreas LipphausLuís Pedro Ferreira RatoAndreas RothLuis RojoAndreas SonessonLuis SantosAndreas StengelLuis VitettaAndrei LucaLuisa JordaoAndrei R. AkhmetzhanovLuis-Alfonso Arráez-AybarAndrej StarcLukas J MotlochAndres BurLukáš PeterAndrés Da Silva-CandalLukasz KrzychAndres MuroŁukasz ŁapajAndrew A. HarrisonŁukasz SobottaAndrew Abba StolzLukasz SzczerbinskiAndrew BahnLuke GrundyAndrew Conway MorrisLuke J. MortensenAndrew DalbyLuke NicholsonAndrew DiNardoLuke OsbornAndrew DixonLulu L.C.D. BursztynAndrew DwyerLuminita A. StanciuAndrew EnglandLunan JiAndrew FarmerLunardi FrancescaAndrew GrayLuxana Reynaga-OrnelasAndrew HighamLuz Angela Torres-de LaAndrew J AschenbrennerLydia E. DevenneyAndrew James WoodLydia H. PeckerAndrew JirasekLydia MederAndrew LandstromLydia VisserAndrew LissLyn O’GradyAndrew M. BlakelyLyn WiseAndrew MackenzieLynissa StokesAndrew Menzies-GowLyudmila I. RachekAndrew Octavian SasmitaM Isabel Torres LópezAndrew TeoM. Amparo F. FaustinoAndrew W. MaksymiukM. Anwar IqbalAndrew WilliamsM. Carmen MontesinosAndric OrtizM. Concetta LupaAndrzej GrzybowskiM. Natalia VergaraAndrzej PolańczykM. Paula MacedoAndrzej SurdackiM. Rosa Bernal-LopezAndrzej SzutowiczM. Teresa Agulló-OrtuñoAndy K.H. LimM.A. FitzcharlesAndy MercerM.F. RaiAnees BahjiM.L. Gonzalez-MartinAneta AleksovaM.P. PeppelenboschAneta BalcerczykM.T. NurmohamedAnette Susanne Bøe WolffMª Dolores Ruiz-FernándezÁngel Álvarez-ArenalMaaike Van PuttenAngel Insua BrandarizMaarten C. BoslandÁngel Oliva Pascual VacaMaarten Van CleeffAngel Romero ColladoMabel ToribioAngela CelettiMacarena DíazAngela Di BaldassarreMaciej BanachAngela FinchMaciej GawęckiAngela GuardaMaciej SałagaAngela M. HeadsMaciej StawnyAngela PearsonMaciej WnukAngela PirilloMaddalena De BernardoAngela Serena MaioneMaddalena MartellaAngela VinturacheMadeline RaveslootAngelica GiulianiMaekawa MasashiAngelo L. GaffoMagdalena DruszczynskaAngelo M CarellaMagdalena DurlikAngie Landeros-WeisenbergerMagdalena Gorska-PonikowskaAnh Nhat TranMagdalena Krajewska-WłodarczykAnindita DasMagdalena KrintusAnissa MoktefiMagdalena LipczyńskaAnisyah RidiandriesMagdalena LuczakAnita JeyakumarMagdalena Paplińska-GorycaAnja BaumannMagnus BäckAnja Kathrin JaehneMahendran ChinnappanAnja VerhulstMahmoud HuleihelAnjali ZarkarMahmoud KhalifaAnkit N. KhambhatiMai RosenbergAnn Helen GaustadMaiBritt GiacobiniAnn M. SchreierMaja Ortner HadziabdicAnn Rita HalvorsenMajed OdehAnn WennerbergMaksym PogorielovAnna AnyżewskaMalathi SrivatsanAnna AtlanteMalgorzata CharmasAnna BaranMalgorzata DobrzyńskaAnna Barańczyk-KuźmaMałgorzata DołowyAnna BartosiewiczMalgorzata KosteckaAnna BersanoMałgorzata LehnerAnna BrooksMalgorzata Maria JerzakAnna CieślińskaMalgorzata SyczewskaAnna DanielewiczMałgorzata WrzosekAnna EsparhamMalgorzata ZakrzewskaAnna GiammancoMalik ZabenAnna Grochot-PrzeczekMalin Lundberg RasmussenAnna K. KurdowskaMalou Van ZantenAnna L. WestermairMalte SgoddaAnna Lena FisseMalte. KornhuberAnna Lisa IorioManabu MochizukiAnna MatyniaManabu MoritaAnna MilesManabu MoriyaAnna MurrayMandy FisherAnna Paola MitterhoferManel SabatéAnna PolewczykManel TaboadaAnna Posadzy-MalaczynskaManfred NeubergerAnna RienmüllerManiselvan KuppusamyAnna StaniszewskaManish MishraAnna Szóstek-MioduchowskaManjunatha Akathatti MunegowdaAnna SzumilewiczManlio VinciguerraAnna V. NaumovaManoj AmrutkarAnna VillariniManpreet Dhuffar-PottiwalAnna Villar-PiquéManu RangachariAnna ZalewskaManuel Albornoz-CabelloAnnabel SpekManuel C. LemosAnnadurai AnandhanManuel Hermida PrietoAnnalicia VaughanManuel Jonas RichterAnna-Maria BarciszewskaManuel Martinez-SellesAnnamaria CarusiManuel Ramírez SánchezAnnamaria CesinaroManuel Saenz De Viteri VazquezAnna-Maria KatsarouManuel SastryAnn-Christin PfeiferManuel ValeroAnne Cathrine ThuesenManuela BonizzoliAnne ChiaramelloManuela PlateAnne R. BassMar LleoAnne Régnier-VigourouxMara BrancaccioAnne S. ReinerMara LeimanisAnnelyn Torres-ReveronMara SimopoulouAnnemarie BaaßeMaralyn FoureurAnne-Marie BouvierMarat EzhovAnnette SchürmannMarc Baud'HuinAnnie NguyenMarc Benjamin HahnAnnie Wong-BeringerMarc Figueras-RocaAnnika ÖhrfeltMarc HilhorstAnnina VischerMarc HuismanAnn-Kathrin StockMarc MelcherAnnunziata RaimondoMarc OriaAnqi ZhangMarc SpielmannsAnthea LindquistMarc Woodbury-SmithAnthony B. MillerMarcello MaddaloneAnthony BonaviaMarcelo NociariAnthony C. FaberMarcin FeldoAnthony GavalasMarcin WozniakAnthony MagitMarco A. CassatellaAnthony VillaniMarco AbbatangeloAntoine LanotMarco Alessandro MinettoAntoine RoquillyMarco BobbioAnton H. Van Den MeirackerMarco BonomiAntonella CotoiaMarco CavaleriAntonella D’AgostinoMarco CicconeAntonella RoettoMarco CirilloAntonello NicoliniMarco ColizziAntoni TorresMarco ColomboAntonia CianciulliMarco CorradinAntonia LockMarco CosentinoAntonietta GiganteMarco Di TolaAntonina GiammancoMarco GaspariAntonino CatalanoMarco HerlingAntonino MignanoMarco InvernizziAntonio C. BiancoMarco LucianiAntonio CarettaMarco MainardiAntonio ChistoliniMarco MaugeriAntonio FacciorussoMarco MeucciAntonio GrecoMarco MongilloAntonio IeniMarco RomanoAntonio Javier Carcas SansuánMarco SardielloAntonio LanzaMarco SebastianiAntonio Magán-FernándezMarco SolcàAntonio Martinez-MillanaMarco TaniniAntonio MazzottiMarco Van LondenAntonio MirijelloMarcos Da SilveiraAntonio NarzisiMarcus KrügerAntonio Palazón-BruMarcus PezzolesiAntonio ScaranoMarcus SchiltenwolfAntonio SerranoMareike LankeitAntonio Simone LaganàMarek BinderAntonios AnastasiouMarek RuchałaAntonios ArvelakisMarek SanakAntony DayalanMargaret Ashley GrevenAnup SinghMargaret E. WiermanAnupam AichMargaret K. YuAnupama ReddyMargareth TzaphlidouAnurag SinghMargarita Corominas RosoAnuska V. AndjelkovicMargarita González-VallinasAnxo Fernández FerreiroMargherita MaranesiApoena RibeiroMargherita TumedeiApostolos BeloukasMargot Van WermeskerkenAppakalai N. BalamuruganMarguerite ClyneAprajita JagpalMaría A. Pérez-HerreroArata SuzukiMaria Addolorata BonifacioArdalan ShariatMaria Adele GiamberardinoArdesheer TalatiMaria Angeles Jimenez-SousaAren BezdjianMaria C. KatapodiAreti StratiMaria Carmen Iglesias-OsmaArheh S. ShanderMaria ContaldoAri MeersonMaria Cristina AisaAri ShechterMaria Cristina D'AgostinoAria NouriMaria Cristina PetraliaArianeb MehrabiMaria Del BenArijit BiswasMaria Del Pilar Acedo NunezArin L. MadenciMaria DigiacomoAristeidis H. KatsanosMaria E. MarketouArjan Van Der TolMaria Elena FerreroArkadiusz GertychMaria Elisa MancusoArmando CaballeroMaria Elisabeth StreetArmando Del PortilloMaria Eugenia Fortes BrolloArmando L. OliverMaría F. Solá-RuízArnab BasuMaria Fiorella SarubboArnab ChattopadhyayMaria Francesca AlvisiArnaud PasquerMaria G. SignoriniArnaud ScherberichMaria GazouliArnon BlumMaria GuarinoArrigo CiceroMaria HernandezArtemissia-Phoebe NifliMaria I. ZervouArthur J. SiegelMaria Irene BelliniArthur LaquiereMaría J López-ArmadaArtitaya LophatananonMaría José García-BarradoArtur KowalikMaría José García-PolaArtur PaivaMaría José Pérez SáezArtur PałaszMaria Kalliopi KonstantinidouArtur SłomkaMaria KambanarosArtur SulikMaria KoukoulakiArturo BevilacquaMaria L. MateusArun KanakkantharaMaria L. PacellaArun SinghMaria L. Sánchez-FerrerAruna ChandranMaria Laura TandaArunava RoyMaria LeeArvind DasariMaria Lorenza MuiesanArvindh N. KanagasundramMaria LucaAsaf AchironMaria Luisa Castro-CodesalAsha KumariMaría M. Morales Suárez-VarelaAsher OrnoyMaria Maddalena AngioniAshish PathakMaria MasulliAshkan EmadiMaría MuriachAshkan Ghanbarzadeh-DagheyanMaria OczkowiczAshlesha KaushikMaria PiccioneAshok MandalaMaría Pilar LagunaAshvin R. JaiswalMaria PolidoriAsnat RazielMaría Roca LlorensAssem G. ZiadyMaría Rosa López-HuertasAssia EljaafariMaria SartoriAston K. McCulloughMaria SwanbergAstrid Kamilla StunesMaria Teresa EspositoAthanasia PapazafiropoulouMaria Teresa MascellinoAthanasios ChalkiasMaria Torres VegaAthar QureshiMaria Werner-WasikAtif ZafarMariafrancesca CascioneAtin A. AgarwalMaria-Giuliana VannucchiAtsunori TstuchiyaMarialaura PetroniAtsushi KameyamaMariam KayleAtsushi OkawaMarian SimkaAttila TordaiMariana MonteiroAtul RanjanMariana S De LorenzoAudrey Cougnard-GrégoireMariana SantosAudrey LongMariane De MontalembertAugustine ChungMariangela AlbertiniAuksė DomeikienėMarianna TorokAurélie RondonMarianne LundAurelija RadzevičienėMarianne SamynAurelio LunaMariano FerraressoAurelio SalernoMariano M LannaAureliusz KolonkoMariapia VairettiAuriel A. WilletteMariasanta NapolitanoAurora VassanelliMarie BillaudAusanee WanchaiMarie C. HansenAustin K. MircheffMarie CarlsonA-W LeeMarie Joelle JabagiAxel HuttMarie LordkipanidzéAxel SchlittMarie Louise Muff WissingAxel SchmutzMarie Noëlle GiraudAxelle Septembre-MalaterreMarie Øbro FosbølAya MousaMarie-Christine SeghayeAya SaitoMarieke GriffioenAyman ElbadawiMarieke M. Ter WeeAzahara Iris RupérezMarij ZuidersmaBaba InusaMarija SajicBabak ChehroudiMarijn SpeeckaertBabette LaMarcaMarijn VisBabken AsatryanMarike W. Van GisbergenBabu Guai WelchMariko Harada-ShibaBahtiyar YilmazMarilyn CyrBalázs KuiMarilyn MoyBalwant S. TuanaMarin VeldicBang-An WangMarina AunapuuBaoli HuMarina Letica CrepuljaBaoyin ZhaoMarina SartiniBapi PaharMarine MondinoBaptiste Couvy-DuchesneMario DioguardiBaptiste LignierMario GalganiBaptiste PignonMario GanauBarak ZafrirMário GrãosBarbara BessetteMario LevisBarbara Bobrowska-KorczakMario MatijašicBarbara GaravagliaMario Perez SayansBarbara Garmy-SusiniMário SousaBarbara Gawronska-KozakMario StanzianoBarbara GivenMariola SłowińskaBarbara LiederMarion T. TurnbullBarbara Ruszkowska-CiastekMarisa SchlichthorstBarbara SotteroMariusz HartmanBarend J Van RoyenMariusz NiemczykBaris KarakullukcuMariusz SikoraBarry L FriedbergMariya MitevaBart PijlsMarjan BoermaBart SpronckMarjan Wouthuyzen-BakkerBart VandenbergheMark A. SperlingBart-Jan KullbergMark D. HicarBartłomiej Henryk NoszczykMark EttenbergerBartłomiej SoltysikMark GibsonBartlomiej ZalewskiMark GorrellBartolomé LlorMark HanudelBasanth Babu EedaraMark HoeggerBassam YaghmourMark J. SoloskiBastian SchirmerMark K. TimmonsBeat GloorMark MiddletonBeáta BőtheMark MolitchBeata HornikMark MuschBeáta HubkováMark PertileBeata Januszko-GiergielewiczMark R Van Der MaasBeata KrasińskaMark SutherlandBeata ŁoniewskaMark TitusBeata SadowskaMark TurnerBeatrice ArosioMark Ulrich GerbershagenBeatrice D'OrsiMark W. BallBéatrice GulbisMarkku TykkyläinenBeatrix BarthMarko KnollBeatriz Martín-AntonioMarkus B SkrifvarsBedros TaslakianMarkus GiessingBee K TanMarkus GraelerBela JuhaszMarkus GugatschkaBela OzsvariMarkus HiltyBelinda BrownMarkus L. LampertBelinda LeeMarkus SchoenbergBellini Maria IreneMarkus W LöfflerBen ChongMarkus W. HollmannBen GibbisonMarnie GracoBen LewisMarquisette Glass LewisBen MulhearnMarshall DevorBen Van HoutenMarshleen YadavBenedetta SallustioMarta Martinez AlonsoBenedetto FalsiniMarta Martín-FernandezBenedetto SacchettiMarta MazurBenedikt LauberMarta MorettiBenedikt SchaeferMarta MoskotBenjamin AnsaMarta PojoBenjamin Arthur TurturiceMarta SupervíaBenjamin B. KastenMartin AasbrennBenjamin KloeselMartin BahlsBenjamin KnierMartin BesserBenjamin OndruschkaMartin BiermannBenjamin VigliantiMartin BlockBenjamin VoellgerMartin HundBenjawan BoonkaewMartin KaukeBenno WeigmannMartin PicardBerenika WebsterMartin ReijnsBerislav BosnjakMartin RutegårdBerit Schiøttz-ChristensenMartin ScharffenbergBernard GenyMartina CebovaBernard HarlowMartina KroppBernard MonclaMartina SeifertBernardetta IzydorczykMartina SolliniBernardo StutzMarty HoffmanBernd StegmayrMarvin DiazBernd WolfarthMarwan A.S. BukhariBernhard HametnerMary Ann SteppBernhard PreimMary Katherine HoyBernhard RauchMary V. SeemanBernhard WernlyMaryam EbadiBernhard ZelgerMary-Elizabeth PattiBert CallewaertMarzena BaranBert GarssenMarzia Di DonatoBert LittleMasa SasagawaBerthony DeslouchesMasaaki MinamiBethany KerrMasaaki MiyazawaBethany L. StanglMasafumi KoshiyamaBethany StanglMasahiko AyakiBetina BiagettiMasahiro BannoBettina EngelMasahiro EriguchiBharani Krishna Mynampati ArunadithyaMasahiro HoriuchiBharat H. JoshiMasahiro KimuraBharat VaidyanathanMasahiro NishimuraBhupesh SinglaMasahiro SugimotoBianka BrunneMasaki FujitaBibiana Garcia-BailoMasaki RiBijan GhalehMasako HaradaBijan K DeyMasanari KuwabaraBiji Theyilamannil KurienMasanori FujisawaBijun ZengMasanori YasuoBill MuskMasaru MiyanoBill WangMasashi MizuguchiBimal ChaudhariMasataka SumiyoshiBin QiuMasataka UmedaBin RenMasato KantakeBinod KumarMasato NakamuraBjoern O. SchroederMasayasu HoribeBjörn GerdleMasayuki OkudaBjörn KemperMasayuki WatanabeBjørn Tore GjertsenMasayuki YamatoBlaise Mathias CostaMassimiliano Di PietroBlake HildrethMassimiliano SorbelloBlanka StiburkovaMassimo CapocciaBob ArgiropoulosMassimo Dal MonteBodil BjørndalMassimo De MartinisBoel De PaepeMassimo FalconiBogdan Andrei SuciuMassimo GirardisBogdan BatkoMassimo IacovielloBojana BeovicMassimo TruccoBoris GuyotMasutaka FurueBoris KotchoubeyMateusz CybulskiBoris VaismanMateusz MaciejczykBouchard StéphaneMathew S. MaurerBradley EdelmanMathews VargheseBradley FergusonMathias RheinBradley McPhersonMathieu JozwiakBrandon McKinneyMathieu Van Der JagtBrandon RobertsMathieu WimmerBrandt PenceMathilde Body-MalapelBrendan GongolMatilde TschonBrenna McDonaldMatjaž ZwitterBrent A. BakerMats HellströmBrent KiousMats W. JohanssonBrian A. BooneMatteo BonatoBrian JacksonMatteo CeredaBrian M. WardMatteo Dal FerroBrian PiperMatteo DonadonBrian R. CurtisMatteo FerroBrian SkaugMatteo GhilliBrian Van NessMatteo LisiBrian W DarvellMatteo MartinolliBrice GouvernetMatteo NeriBridget E BaxMatteo Nicola Dario Di MinnoBright Senyo AshimateyMatteo RespinoBrigitte HantuschMatthew BrowneBrigitte Le Magueresse-BattistoniMatthew D BensonBrigitte LitschauerMatthew D.F. McInnesBrigitte WilczekMatthew Fuller-TyszkiewiczBriony HillMatthew HallBrittanie LockardMatthew J BartonBritt-Isabelle BergMatthew McMillinBrock HumphriesMatthew RätsepBruce BauerMatthew RobertsBruce KimlerMatthew S. PihlbladBruce RothschildMatthew SchumannBruna SinjariMatthew SouthBruno Bernardes De JesusMatthew SutherlandBruno ChrcanovicMatthew T.s. TennantBruno ContiMatthias BülhoffBruno EllaMatthias ClaussBruno FantinMatthias D. HoferBruno FattizzoMatthias DerwallBruno HerbelinMatthias KapischkeBruno MarinoMatthias KapplerBruno MinottiMatthias KnobeBruno MoncharmontMatthias VogelBruno Ramos-MolinaMatthieu RuizBruno TrimarcoMaureen LeonardBryan CrowMaurice LingBryan S. BennMaurits H.T. De RuiterBryan YoungMaurits Van SelmsBulang HeMaurizio AcampaBungo OtsukiMaurizio Battaglia ParodiByoung-eun YangMaurizio BonatiByung Kook LeeMaurizio EliaByung-Wook KimMaurizio GiulianiC (Johnny) ChenMaurizio InghilleriC. LenschowMauro CancianCai-Mei ZhengMavis TenkorangCaitlin KeighleyMax Christoph LiebauCalin HomorodeanMax E JoffeCalogera PisanoMax MasthoffCamelia DiaconuMax PaquetteCamila Martinez CalejmanMaxim MoreauCamilla HageMaxime MaheCamilla Kin Ming LoMaxime PichonCamilla MaccarioMaximilian KlarCândida Teixeira TomazMaximilian SallerCaren J. FrostMaximilian Sebastian SchaeferCarl M. PhilpottMayuko Osada-OkaCarla CaffarelliMayumi NakagawaCarla CicchiniMayura MeerangCarla ComacchioMaziar Ashrafian BonabCarlo BuonerbaMd Kamal HossainCarlo CervellatiMD. López-CarmonaCarlo ChizzoliniMechthild Westhoff-BleckCarlo DoriaMedha AIryCarlo FornainiMee Kum KimCarlo GalliMegan E. MaroneyCarlo LajoloMegan Horn OrzalliCarlo MaioranaMeghan McGee-LawrenceCarlo PerriconeMehmet OzenCarlo RodolfoMeiyun FanCarlo SaittaMelanie J. ChandlerCarlo TicconiMelissa A. St HilaireCarlo VigoritoMelissa L ConstantineCarlo ViscomiMelissa MeadeCarl-Olav StillerMellar P. DavisCarlos E. VelascoMengyang LiuCarlos Pérez-SánchezMeng-Yu WuCarlos Plaza-SirventMercedes MarinCarlos Santos-GallegoMercedes MarínCarlos StoccoMeredith K. GinleyCarlota SaldanhaMeta N. EekCarlotta PalumboMette D. HazenbergCarlotta PipoloM-F González-EscribanoCarmela CaputoMiao-Fen ChenCarmela NardelliMicaela GliozziCarmela Rita BalistreriMicah ZuhlCarmelo BellarditaMicha Tobias MaederCarmen CadillaMichael A KalwatCarmen Escobedo-LuceaMichael A. KennedyCarmen RequenaMichael A. NeadCarmen Rodriguez-CuetoMichael A. RamsayCarmine OnofrilloMichael A. ViaCarol K. ChanMichael AradCarola Van DiptenMichael C BurgerCarolina SimioniMichael CangkramaCaroline M. VassMichael CasaerCaroline N. DealyMichael CatapanoCaroline WachtlerMichael CrossCarolyn BerrymanMichael D. MaileCarolyn CestaMichael D. PerloffCarolyn Drews-BotschMichael E BrierCarolyn J. GreeneMichael EdmondsCarrie FinnoMichael FehlingsCarrie GraveelMichael FroelichCarrie McAdamsMichael FurianCarrington ReidMichael H. AntoniCarsten HaferMichael HoaneCary A. BrownMichael IosifidisCassio RuasMichael J. CoffeyCatalin VasilescuMichael J. NotarasCatarina Maria QuinziiMichael J. VoorCatarina RamosMichael KlontzasCatarina Sousa GuerreiroMichael KunzeCaterina AgrimontiMichael KurzCaterina MottaMichael LangCatherine CoombsMichaël LaurentCatherine Margaret KuzaMichael LichtenauerCatherine MorganMichael MsallCatherine PaillardMichael Noll-HussongCatherine PenningtonMichael P. AngaroneCatherine St HillMichael R. PreuschCatherine ViguiéMichael RobesonCatherine Woodstock StrileyMichael RotherhamCathryn CrowleMichael SchaeferCátia CaneirasMichael SmoutCatia CillonizMichael T. KoltzCecelia CalhounMichael TannerCecilia ManciniMichael TengCecilia VecoliMichael UlbigCedric HermansMichael WilsonCees A. SwenneMichael ZaretskyCelestino RodriguezMichael ZileCelestino SarduMichaela TencerovaCeline BerthierMichał BijakCesar Ariel KellerMichał BorysCésar Calvo LoboMichal CiborowskiCésar Calvo-LoboMichal CiebieraCésar Hueso-MontoroMichal Katz-LeurerCesar M. CastroMichal KawulokCesar Salinas-La RosaMichał KunickiCesare CuspidiMichał NowickiChad PopeMichał PędziwiatrChan Young ShinMichal PolgujChandni Maria JacobMichal ŠteffChandrakanth EdamakantiMichał WaszczykowskiChandramohan WakadeMichał ZarobkiewiczChandran NagarajMichel JadoulChang Myeon SongMichela BattistelliChang Seok BangMichela TerlizziChang-il HwangMichele AmmendolaChang-Nim ImMichele ColledanChang-Sei KimMichele CorrealeChangwan HongMichele Di MauroChang-Wen ChenMichele DonatiChanpreet SinghMichele F. EisengaChao ChenMichele GhidiniChao RongMichele LorenzoCharalampos PontikoglouMichele LuchettiCharat ThongprayoonMichele PaganoCharikleia StefanakiMichele RoccellaCharlene W. CompherMichele StoccheroCharles D. BurgerMichele VitaccaCharles E. WadeMichelle ArmstrongCharles GuenanciaMichelle OlaitheCharles J. GlueckMichelle PalmerCharles N. CornellMichelli Faria De OliveiraCharles S KamenMichihito KyoCharles W. LanksMickey LanglaisCharles W. SchindlerMieczyslaw SzyszkowiczCharlotte BeaudartMieke A. SoensCharmion Cruickshank-QuinnMieszko WieckiewiczCharol ShakeshaftMiguel Angel Martinez GarciaCharvi NanavatiMiguel Ángel MorcilloChary López-PedreraMiguel CarreiraChasity M. SheltonMiguel de Araújo NobreCheah ChanMiguel HuesoChen KangMiguel LeãoCheng JiangMiguel Padial-MolinaCheng-Chang ChangMiguel PenarrochaCheng-Chung WeiMiguel Silva VieiraCheng-Fang YenMiguel Souto BayarriCheng-I ChengMihaela Ileana IonescuChengying HsiehMihaela ZegreanChen-Hwan CherngMihai Dorin VartolomeiCheorl-Ho KimMihnea-Ioan NicolescuCherie P. ErkmenMiin RohCheryl A. ConoverMika L. NonoyamaCheryl A. WinklerMikael J. TurunenCheryl BrunelleMikalai M. BudzevichCheshta SharmaMike BarbeckChetna SoniMike ShoemakerChewei WuMike YangCheyenne NewsomeMikhail MaslovChi ZhangMiki UchinoChi ZhouMikk JürissonChia-Jung LiMiklos Sahin-TothChia-Liang TsaiMilagros MedinaChiara AngiolilliMilena SokolowskaChiara AttanasioMiles A. MurphyChiara BerteottiMiles WeinbergerChiara CencioniMilica VukmirovicChiara DemartiniMilka KoupenovaChiara DiquigiovanniMin ZhaoChiara EandiMingfeng BaiChiara GamberiMing-Horng TsaiChiara GiacomelliMing-Ju TsaiChiara LazzeriMing-Ju WuChiara PlataniaMing-Ko ChiangChiara SaponaroMingKuan SUNChia-Siu WangMing-Shao TsaiChia-Ter ChaoMing-Tse KuoChia-Yu ChiMinhhuyen T. NguyenChien-Feng LiMin-Seong HaChien-Hung LeeMira BarakChien-Kun TingMiranda Nicole ReedChih-Ching WuMiranda Van LunterenChii-Ming LeeMireia TondoChing-Chi ChiMirela FrandesChing-Chi LeeMiriam HoeneChing-Hua HsiehMiriam SchiffChing-Li TsengMiriam SchmidtsChirag GajjarMiriam T. LevyChi-Tai YehMiriam ZacchiaChitra IyerMiriam-Rose MenezesChitra UpadhyayMirjam KeßlerChiu-Ming HoMirosława Ferens-SieczkowskaChloe LaneMirto FolettoChloe S. SlocumMitsushige SugimotoChris Platania-phungMitsuyo KinjoChris ShidalMitsuyoshi TakaharaChrista LabouliereMizuho NishioChrista LohrmannMoeezullah BegChrister DahlinMohamad HabesChristian A. GutschowMohamad SalkiniChristian Alexander BehrendtMohamed AbbasChristian BesteMohamed Abdel-NasserChristian CarulliMohamed EtafyChristian E. BryantMohamed GhorbelChristian FischerMohamed O. OthmanChristian HydeMohammad AbufarajChristian JungMohammad AsimChristian KonradsMohammad DerakhshanChristian LehmannMohammad Zakir HossainChristian LinzMohammed Abdul-GhaniChristian MontagMohammed F FaramawiChristian OsadnikMohammed S SalahudeenChristian PilarskyMohan RudrappaChristian RoquesMohanraj SadasivamChristian SelingerMohd Parvez KhanChristiana KartsonakiMohtashem SamsamChristiane MühleMollie MonnigChristina HolzelMolly Scannell BryanChristina KruuseMona BatishChristina L EkegrenMona HaronChristine Attenhofer JostMona MomeniChristine BojanowskiMona StåhleChristine DetrembleurMonica AcostaChristine HeberdenMonica Cartelle GestalChristine McDonaldMonica GorassiniChristodoulos E. PapadopoulosMonica Mattioli-BelmonteChristof LeichtMonica MottesChristoph IhleMonica ValentovicChristoph KahlertMonika HaackChristoph KesselMonika HertenChristoph MitschMonika KlimkowskaChristoph NienaberMonika Lukomska-SzymanskaChristoph ReinhardtMonika RatajczakChristoph SinningMonish Ram MakenaChristoph TabelingMontse MercadéChristophe O. SoulageMontserrat Díaz-EncarnaciónChristophe SoulageMontserrat SallerasChristopher BliemelMonty SilverdaleChristopher CossMoran AmitChristopher David TurnbullMorayma ReyesChristopher G. WilsonMorena ShkodraChristopher J RomeroMoreshwar DesaiChristopher J. A. DuncanMoritz WildgruberChristopher J. WinfreeMorten DahlChristopher M. OlsenMorten HostrupChristopher M. ReillyMorten QuistChristopher ManssonMosiewicz JerzyChristopher P. KovachMostafa BakhtiChristopher R. CederrothMoussa Antoine ChalahChristopher R.J. WoodhouseMrinal K. SarkarChristopher S. HaywardMrinmoy SanyalChristopher StubenrauchMuhammad Faraz MasoodChristopher ToweMuhammad SaeedChristopher W CutlerMuh-Hwa YangChristos ArgyropoulosMukundan RagavanChristos ChatzikyrkouMuna T. CanalesChristos MikropoulosMunish ChauhanChristos TikellisMuppala N. P. RajuChuan LiMurali YandaChulhun L. ChangMustafa OzenChul-Su YangMuthukumar KannanChunbin ZouMuy-Teck TehChung How KauMy HedhammarChung Y. HsuMyriam RubioChung-Wei YangMyriam ThomaChun-Hua WangN. BakeerChunhuan LaoNa ZhuChun-Nan YehNabin PaudelChunyu LiuNadeeja WijesekaraChu-Yu LeeNadia AlfaidyCidália Dionísio PereiraNadia NajafiCinzia AntognelliNadia NathanCinzia MasperoNadim MahmudCiro ConversanoNadine HornigCiro CostagliolaNadir YehyaCiro ManzoNagaia CiacciClaire ConleyNagarjuna Reddy CheemarlaClaire MorganNagib DahdahClaire WhyteNaiara DemnitzClara De PalmaNai-Jen ChangClarissa BerardoNajat SalamehClaudette PooleNajib T. AyasClaudia A. BlindauerNakamura TaishiClaudia CavaNalini M RajamannanClaudia CohnNamkoong HoClaudia CrociniNancy GarofaloClaudia GüntherNancy OleinickClaudia Rangel-BarajasNancy S. GreenClaudia RicciNancy W GlynnClaudio AndreettiNanda Van Der StapClaudio BucoloNandor Gabor ThanClaudio De LuciaNans FlorensCláudio MaiaNaoki HasegawaClaudio StacchiNaoki IwamotoClaudio TanaNaoki NishidaClaudio TomaNaoki UmemuraClaudio TorresNaoko NakamuraClaudio VergariNaoko YanagisawaClaudiu BandeaNaoro TanakaClaus VoglNaoto KubotaClemens DuerrschmidNaoya MasumoriClemens FürnsinnNaoyasu YoshidaClemens JilekNapoleon WaszkiewiczClemens WittenbecherNardelli CarmelaColin P. ChurchwardNaro OhashiColin PurdieNarothama Reddy AeddulaColleen CronigerNarut PrasitlumkumColleen D. PeytonNatália BarbaroCombarnous YvesNatalia BudaConcepción Aguilera GarcíaNatalia InadaConcepción López-SolerNatalia MalloCongcong YinNataša Vidovič ValentinčičConglei LiNatasa ZenicCoralie GandreNatascia TisoCord LangnerNatasha A. KhovanovaCORINA LORZNatasha KyprianouCorina Marilena CristacheNatasha SigalaCorinne PondarréNathalie AugerCornelia ThenNathalie StakenborgCorrado PiconiNathan LanningCostantino LeonardoNathan Peiffer-SmadjaCostas FourtounasNathanael J. YatesCourtney FitzhughNatividad BenitoCourtney L. ScaifeNaveen SinghCourtney ShellNavneet BaidwanCraig Stephen WebsterNavrag SinghCreed Michael StaryNazareno PaolocciCrescenzio SciosciaNazia ChaudhuriCrista B. WadsworthNazita TaghaviCristian FioriNeal ParikhCristian MornosNeeraj ChauhanCristiana CiprianiNeerupma SilswalCristina Adela IugaNegar ManavifarCristina HernandezNeha BhatCristina MaccalliniNehal A. ParikhCristina P. RodriguezNeil C. ThomsonCristina SoléNeil G SimonCristina TognonNeil G. DochertyCristina TravelliNeil P. OxtobyCristina UlivieriNeil SmartCristina VinciNejat DuzgunesCristina-Mihaela LăcătușuNeli Kachamakova-TrojanowskaCristoforo ComiNelson YeeCsaba MattaNiccola FunelCuong Hoang-VuNicholas BradshawCynthia J. Koziol-WhiteNicholas FuggleCynthia KarlsonNicholas ObermüllerCynthia L LancasterNicholas RowanCynthia SprengerNicholas T. BelloCyril Le NouenNicholas TentolourisCyril MoersNiclas LampertCyril PernetNico De VriesCyrus MotamedNicola Alberto ValenteDa Silva DanielNicola BerlandaDa Young OhNicola FerriDag NymanNicola HeneghanDag SehlinNicola Ivan LorèDagmara JaworskaNicola LambertiDaiji KawanamiNicola MaffulliDaimantas MilonasNicola MumoliDaiqing LiaoNicola NanteDaisuke FujikuraNicola PetrosilloDaisuke KodamaNicola RotoloDaisuke NakashimaNicola SalvadoriDaisuke SanoNicola StrisciuglioDale QuestNicolaas A.P. FrankenDalia AdukauskienėNicolai GoettelDalila Laoudj-ChenivesseNicolai Jacob Wewer AlbrechtsenDalius JatužisNicolai KroghDamain HolsingerNicolas A. KarakatsanisDamaris RohsenowNicolas BoisgeraultDamián GuiradoNicolas J. DedyDamian RyszawyNicolas PineaultDamiano TaniniNicole DelimontDamien A. LeachNicole GattoDamien HarkinNicole K RendosDamien LannoyNicole KarcherDamien RiggsNicole WilliamsDamien ViglinoNiek H. PrakkenDamir HudetzNiels HellingsDana Maria CopoloviciNiels Nørskov-LauritsenDana MunteanNiels Vande CasteeleDana N. RaugiNiema PahlevanDana SteidtmannNihal El RoubyDana StoianNikhil JoshiDana YagilNiklas EngströmDandan GuoNikola SaulacicDandan ZhuNikolaos GatselisDanguolė RugytėNikolaos V. MichalopoulosDaniel A. BaroneNikolas HeroldDaniel Alonso-AlconadaNikolaus HaasDaniel ÁlvarezNikoletta RovinaDaniel B HortonNilisha FernandoDaniel CampbellNino RussoDaniel CohenNir PeledDaniel De La IglesiaNirav H. AminDaniel E BauerNirmal VermaDaniel F. KellyNirvedh MeshramDaniel González-NietoNisha DurandDaniel GrinbergNisha NairDaniel HackettNishanth E. SunnyDaniel Hernandez-VaqueroNissreen ElfadawyDaniel HertzNithin B. BoppanaDaniel JohnsonNitin KumarDaniel K. JeongNivee P. AminDaniel KetelhuthNoain DanielaDaniel L. HertzNoboru SuzukiDaniel LópezNobuhiro MatsumotoDaniel López-LópezNobuhiro NakamuraDaniel M. FeinNobuo TomizawaDaniel M. HarrisonNoelia Muñoz-FernándezDaniel NationNoman NaseerDaniel R. MachinNorbert Joachim TripoltDaniel RamsköldNoreen RossiDaniel SauterNoriko KinoshitaDaniel ScottNuno Araújo-GomesDaniel SinnettNuno RaimundoDaniel TszeNunzia Di MaggioDaniel TurnerNuria Álvarez-SánchezDaniel WendlingNuruddin UnchwaniwalaDaniel WestNyaz DidehbaniDaniela Acquadro MaranOdunayo MugishoDaniela CabibiOfer ReiterDaniela CarnevaleOfira EinsteinDaniela DamianiOh-Kyeong KweonDaniela De FilippoOHNO NobuhikoDaniela Filipa Duarte CamposOka KyokoDaniela M. DinulescuOke GerkeDaniela PiancatelliOla StenqvistDaniela QuaglinoOlaia Martínez IglesiasDaniele BaniOlatz ArteagaDaniele BellaviaOlav W. NielsenDanielle N. DesautelsOleh AndrukhovDanielle ShpinerOleksa RewaDanijela PiskulicOlena RudykDanilo BadialiOlga KislitsinaDanuta Januszkiewicz-LewandowskaOlga ScudieroDaria IlatovskayaOlga SukochevaDario BrunettiOliver HirschDario BruzzeseOliver KönigsbrüggeDario RuscianoOliver KripfgansDario SiniscalcoOliver StrobelDarius SoonawalaOlivia CarterDariusz ŁątkaOlivia MaynardDarren LeeOlivier Alexandre LucarDarshan TrivediOlivier DormondDaryl JonesOlivier DutourDavid A. C. SimpsonOlivier MimozDavid AbbottOlle RingdénDavid ArcherOluf AndersenDavid Bar-OrOmar CauliDavid BentleyOmar M. Al-JanabiDavid BerbrayerOmbretta RepettoDavid BradleyOmonigho BubuDavid BruceOriela RustemiDavid C. IrwinOsama E. RahmaDavid CassimanOscar BartulosDavid D'AndreaOscar CampuzanoDavid DaviesOscar González-MartínDavid De GonzaloÓscar Lorenzo GonzálezDavid DunckerÓscar PozoDavid E. JaramilloOtto PhanstielDavid FindlayOtto SanchezDavid GraceyOxana KlementievaDavid H. WagnerOyekoya AyonrindeDavid HallfordPablo Cervera-GarviDavid HallmanPage AndersonDavid Hervás-MarinPål Kristian SelboDavid HewsonPaloma Mari-BeffaDavid KeanePalsamy PeriyasamyDavid KomatsuPamela SenesiDavid L. WrightPanagiotis LainasDavid M MaslovePanagiotis MallisDavid M. DudzinskiPanagiotis PaliogiannisDavid M. Lydon-StaleyPanagiotis PeitsidisDavid MazerPaola Di PietroDavid MorrisPaola FerrariDavid NiederseerPaola PalumboDavid ParrPaola PontrelliDavid PaulPaola QuarelloDavid PejoskiPaola RomagnaniDavid Rhys AxonPaolo A CortesiDavid Rodríguez SanzPaolo AlbertonDavid Rodríguez-SanzPaolo BoffanoDavid RosenbaumPaolo CameliDavid Ryan OrmondPaolo CapparèDavid ScottPaolo CavorettoDavid Scott McVeyPaolo GhislettaDavid StegnerPaolo GiuffridaDavid StephensenPaolo MartellettiDavid TurnbullPaolo NucciDavid VaudryPaolo PesceDavid WhitcombPaolo PoggioDavid WoodPaolo PreziosaDavid ZweikerPaolo RiccioDavide BarbagalloPaolo RuzzaDavide CampanaPaolo SeverinoDavide CampocciaPaolo Usai SattaDavide ChiumelloPär HedbergDavide CucchiPär JohanssonDavide EliaParamdeep BilkhuDavide FarronatoPascal EscherDavide Giuseppe RibaldonePascal H.G. DuijfDavide Maria CammisuliPasquale De FranciscisDavide RibaldonePasquale PisapiaDavide ViggianoPatric DelhantyDa-Wen LuPatrice DeckerDawid BączkowiczPatrice E. FortDawid StaudacherPatrice ForgetDawn GulickPatricia A. J. MullerDazhong XuPatricia A. ShiDe Vries Linda SimonePatricia BroderickDean G KaralisPatricia Fernandez-RobredoDean HarrisPatricia J. McLaughlinDean PiconePatricia K. CoyleDeanna L PlubellPatricia KhashayarDebbie SnellPatricia LiWangDebopam SamantaPatrick BradshawDeborah RudinPatrick D. W. KielyDeborah RutmanPatrick De BoeverDeborah WilliamsPatrick D'HaeseDeepak ParasharPatrick DohertyDeepak RainaPatrick G. ArndtDeipanjan NandiPatrick GeraghtyDejan NikolicPatrick HaubruckDell HoreyPatrick HughesDemin CaiPatrick K. BenderDeng-Ho YangPatrick MüllerDenis CorbeilPatrick NasarreDenise TaylorPatrick OwenDeniz HosPatrick T. McGannDennis GörlichPatrizia CancemiDennis KimPatrizia DianaDennis MeestersPatrizia FerroniDerek TomsPatrizia GiannoniDerk FrankPatrizia PignattiDer-Sheng HanPatrizia SuppressaDer-Yuan ChenPatrycja Nowak-SliwinskaDesmond YapPatryk RzońcaDespina D. BrianaPaul BatchelorDespina SanoudouPaul BlochDeutsch DalePaul CarrierDevasmita ChoudhuryPaul GardDevayani BhavePaul GastonDeven PatelPaul J YongDhiraj KumarPaul Li-Hao HuangDhruba PathakPaul MarikDhrubajyoti BandyopadhyayPaul MearaDi LangPaul Michael RamirezDiana BondermanPaul MossDiana GutsaevaPaul RiskaDiana JalalPaul S. PagelDiana MieliauskaitėPaul SundaramDiana ThomasPaul VaucherDiana TilevikPaul W M FedakDiana Van Lancker SidtisPaula HoffDiane M. DaubertPaula Roszczenko-JasinskaDidier F. PisaniPaulina DumnickaDiego CazzadorPaulina Galka-MarciniakDiego García-AyusoPaulina Niedźwiedzka-RystwejDietmar H. SpenglerPaulina SamczukDietrich A. RuessPauline GulliverDimitra NikoletouPavan Kumar PrathipatiDimitrios DionysopoulosPavel MichálekDimitrios I. AnyfantakisPavel RozsivalDimitrios KiortsisPavel SoučekDimitrios NasioudisPawel Antoni KolodziejskiDimitrios P. BogdanosPaweł GacDimitrios Terentes-PrintziosPaweł GaćDimitrios TziakasPaweł Petkow-DimitrowDINA DI GIACOMOPaweł ReichertDina HingoraniPaxton V. DicksonDinesh GundapaneniPayel SilDinesh JillellaPaz Pérez-VázquezDipa PatiPE CippàDirk BaierPedro CostaDirk CysarzPedro Mendes-BastosDirk EgginkPeeyush N. GoelDirk SmitPegah KhoshpouriDirk Von LewinskiPeijun GongDivella RosaPengcheng WangDivya BhagirathPenghong GuoDjuro KosanovicPeng-Hui WangDmitriy M. NiyazovPentti TengvallDolores Garcia-GimenezPentti TienariDomenico Azarnia TehranPer KarlssonDomenico De BerardisPerry GrigsbyDomenico IntisoPeter AhnertDomenico MavilioPeter B. GrayDomenico PaparellaPeter BencsikDomenico RomeoPeter Edward McKennaDomingo Orozco-BeltránPeter EuDominik BeckPeter F. MayeDominik BuckertPeter FrykholmDominik RachońPeter HauDominik SaulPeter HeiduschkaDominika M. PindusPeter I. MilnerDominique LAMARQUEPeter J. ThompsonDon G. MorrisPeter JohansenDon PoldermansPeter K. PanegyresDonald AbramsPeter Kerr HenkeDonald S. ProughPeter L. WhitesellDonald StevensonPeter M. BurckhardtDonato CalabriaPeter N. SchlegelDonato D’AgostinoPeter PensonDong Ah ShinPeter PlomgaardDong Gui HuPeter QuinnDong Jun LimPeter SchemmerDong Oh KangPeter WetselaarDong ZhouPeter WhittakerDongqing YanPeter WitzgallDongshan ZhuPeter. C TaylorDongya JiaPetr G. VikhorevDongyu JiaPetr StarostikDoreen SchmidlPetr TvrdikDorina LauritanoPetra LiskovaDoris VandeputtePetra MelaDorota FormanowiczPetros PapadopoulosDorota H Szczesna-IskanderPetros SyrrisDorota KregielPhil G. CampbellDorota Oszutowska-MazurekPhilip A FriedlanderDorota Siwicka-GierobaPhilip LindnerDorothée FaillePhilip R. MuskinDorothy A NelsonPhilip R. StanforthDoug SidellPhilip W. S. NewallDouglas BorchmanPhilipp BaumeisterDouglas C. HooperPhilipp EllerDouglas David GunzlerPhilipp EnghardDouglas W. LosordoPhilipp KlaritschDouglas WardPhilipp LeuchtDragan PrimoracPhilipp Meyer-MarcottyDragana GabrićPhilippa FibertDragomir MilovanovicPhilippa SaundersDrucilla J. RobertsPhilippe Brunet De La GrangeDuarte MarquesPhilippe JawinskiDubravka Švob ŠtracPhilippe MarteauDuc-Hiep BachPhilippe R KoninckxDuo ZhangPhilippe RigoardE J De BruinPhilippe. MervielE. Mark WilliamsPhillip MüllerE.K. KapsogeorgouPhillip NewtonEberhard LurzPia Lopez-JornetEberhard RabePiera VersuraEberhard UhlPierachille SantusEckart HanekePierangela TottaEddie EbramzadehPiergaspare PalumboEddy J. DavelaarPiergiorgio MessaEdgar Ramos VieiraPiergiuseppe AgostoniEdith ClarosPierluigi Di SebastianoEdmundo Rosales-MayorPiero AmodioEdna PatatanianPiero PavoneEdoardo SciattiPierosandro TagliaferriEdoardo StaderiniPierpaolo TrimboliEdomer TassonyiPierre BoriesEdouard TuaillonPierre DuquetteEdris WediPierre SaintignyEduard Pedemonte-SarriasPierre SingerEdward James Frazer DansonPierre SujobertEdward NęckaPierre-Jean LamyEdward RabyPierre-Marie LepretreEdward SchwarzPierre-Yves RobillardEdwin ArokePieter MartensEdwin J. BoezemanPietrantonio RicciEdwin LimPietro A. ModestiEdyta BarnasPietro CaironiEdyta ReszkaPietro NapoliEffrosyni ManaliPietro RuggieriEfstathia PapadaPiia KarisolaEfstathios MichalopoulosPilar AlcaideEfthimios DeligeoroglouPinaki DuttaEgbert OosterwijkPinar Uysal-OnganerEglė KontrimavičiūtėPing Chih HsuEgle MiliaPing-Fang ChiuEhtesham ArifPinky Budhrani-ShaniEidi ChristensenPiotr CeranowiczEiichi SatoPiotr FormanowiczEiichi WatanabePiotr KozlowskiEiichiro SandoPiotr LadyzynskiEija KönönenPiotr PoplawskiEike SteinmannPiotr SkrzypczykEiki KoyamaPiotr WilczekEinar ArnbjörnssonPiotr WlaźEisenberg Dan TPoli GuidoEishi AshiharaPolychronis KostoulasEisuke AmiyaPooja TeotiaEl˝od Ern˝o NagyPooja TiwariElbert AlmazanPooyan MakvandiEleanor RiveraPoulami DattaEleanor TownsendPrabaha SikderElena BarbieriPrabhatchandra DubeElena CasiraghiPradeep Kumar SacitharanElena CicheroPradeep V. KadambiElena CriscuoloPradeepkiran Jangampalli AdiElena CsernokPraful R. NairElena G. BignamiPragney DemeElena IzkhakovPrajna P. GuhaElena JonesPrakash ChandraElena Niculina DragoiPrakash KshirsagarElena PiccininPrakash RaiElena RampanelliPramod K. GuruElena ReddiPranay SrivastavaElena ReynosoPrasad DandawateElena RinaldiPrasad RallabhandiElena RizaPrasad ShirvalkarElena TombaPrashant NighotEleni AndreouPrashant SakharkarEleni K. DouniPrashant ThakkarElettra MerolaPrashanth AnamthathmakulaElia ArmelliniPrashanth RawlaElias B. RizkPrathab Balaji SaravananElias K. SpanakisPratibha SharmaEligijus PoškusPratik ChhatbarElio MazzonePraveen K. ChandrasekharanElisa ArdizzoniPraveen Ramakrishnan GeethakumariElisa AssirelliPreeti BharajElisa BallardiniPrema Latha MallipeddiElisa BelluzziPřemysl MladěnkaElisa CairrãoPrintza AthanasiaElisa Di PasqualePriscilla G. WittkopfElisa GenuardiPritish MondalElisa OltraPritmohinder S. GillElisabet RothenbergPriya BalasubramanianElisabeth EppardPriya LuthraElisabeth Nieveen Van DijkumPriyadarshini PanthamElisabeth Sornay-RenduPriyadharshini DevarajanÉlisabeth TraiffortPriyanka KulkarniElisabetta AldieriPriyanka VermaElisabetta BalestroProgyaparamita SahaElisabetta CostantiniProsper N'GouemoElisabetta DonzelliPrzemyslaw J. KotylaElisabetta IacopiPrzemysław KrakowskiElisabetta LiveraniPrzemysław LisińskiElisabetta VersinoPrzemysław Tomasz ParadowskiEliška SovováPuneet BajajEliza A. HawkesPurushottam LamichhaneEliza C. MillerPushpa Raj JoshiEliza J.R. PetersonQi TanElizabeth C AkamQi YuanElizabeth K. LundQian ChenEllen KemlerQian ZhangEllen RocheQifan ZhuEllen-Margrethe HaugeQifeng HanEllie Lawrence-WoodQin Xiang NgElmar AignerQingbin CuiElnaz YaghiniQingdong GuanEloisa Guerrero BaronaQingen MengElsa LamyQiuyu ZhuEl-Sayed M AbdelwhabQuirino LaiElse Marie BartelsR. Rivkah IsseroffElske BosR.J.H. CustersElvira De Luna-BertosRachel G. GreenbergElyse Cornett BradleyRachel L O’DonnellElżbieta Łodyga-ChruścińskaRachel LenhartEmanuela BottaniRachel PopeEmanuele BerardiRachel WallerEmanuele RinninellaRacquel Domingo-GonzalezEmer Van RyswykRadica AlicicEmerito Carlos Rodriguez-MerchanRadovan BilekEmiel F.M. WoutersRadovan Slezákemilie narni-manchinelliRadu Paul MihailEmilio González-ReimersRaelene PickeringEmily K. TarletonRafael A. Caparros-GonzalezEmily NewmanRafael CarbonellEmily PadhiRafael Delgado RuizEmir BencaRafael Delgado-RuizEmma H. BakerRafael GolpeEmma J.M. Barinas-MitchellRafael Y. LefkowitzEmma MotricoRaffaela PeroEmma PerssonRaffaele GiordanoEmma WallaceRaffaele PalladinoEmmanuel CharbonneyRaffaella CancelloEmmanuel GabrielRaffaella ColombattiEmmanuel KatsanisRaffi GugasyanEmmanuel ProkopakisRahul KashyapEncarnación G. CarreteroRaimondo Maria PavarinEng H. OoiRaina GergovaEng OoiRainer H. A. FinkEngy T. SaidRainer ReileEnhad ChowdhuryRaj PadwalEnitome E. BaforRajashekhar GangarajuEnke BaldiniRajdeep DasEnric ReverterRajendra K GangalumEnrico AmicoRajesh ParsanathanEnrico BorrelliRajib MukherjeeEnrico BroccoRajinder DawraEnrico CarminaRajit K. BasuEnrico FiaccadoriRajni GoyalEnrico FioriRakesh BhattacharjeeEnrico MoroRalf DörnerEnrico RizzarelliRalph TrippEnrique Gómez-BarrenaRam PrasadEnrique J. Gallego-ColonRama JayarajEnyuan LinRamandeep S. TakhterEnzo IerardiRamesh KasettiEphraim WinocurRamesh KumarEphrem Takele ZewdieRamesh PeriasamyEray YagmurRamesha PapannaEric C. BurtonRami A. Al-HoraniEric Christopher ShattuckRamon GuiradoEric L. SmithRamona GalatusEric LevesqueRamūnas UnikasEric M. SarpongRamy M. HannaEric S. HoRamzi M. MohammadEric ShymanRan JingEric TruyRan NeigerEric YoshidaRandall E HarrisErica Cherry KemmerlingRandall J. RoperErik A. BillingRANJEET DASHErik BehringerRanjeev Chrysanth PulleErik DomellöfRanjit DeshpandeErik L. GroveRaoul SagginiErik OudmanRaphaël MaréeErika CecchinRaphael P.H. MeierErika Di ZazzoRaphael RoduitErika Fernandez-VizarraRaphael SchusterErika GucciardoRaquel L. BernardinoErin E. ButlerRaquel Mayordomo AcevedoErin F. BarretoRatko DjukanovicErin J HowdenRatnadeep MukherjeeErin Nesbitt-HawesRatnakar SinghErin RobinsonRaúl RecioErin WestgateRaul S. GonzalezErlend.Johan SkraastadRaúl Tárraga-MínguezErnest U. EkpoRaúl Teruel-MontoyaErnesto Cortés CastellRaul Zamora-RosErwin (W.E.) Van SpilRavinder KaundalEstate SokhadzeRavindra K. PandeyEsteban L. PorriniRay W. SquiresEsteban Orenes PiñeroRayaz A. MalikEsteban Orenes-PiñeroRaymond Scott TurnerEsterina PascaleRebecca ColmanEsther T. MaasRebecca G. RogersEswar ShankarRebecca GraffEthan BenardeteRebecca GreenEthan K MurphyRebecca L. HeiseEttore BidoliRebekka HeitmarEttore CaroppoRecep AvciEufemia JacobReda MahfouzEugene Haydn WaltersReeta LamminpääEugene HwangRegina GrahamEugene LinRegis BobeEugene YangReidar P. LystadEugenia AllegraReinhard W. KösterEugenia MatoReinier TimmanEugenio MartelliReinstadler SebastianEugenio PedullàRejeesh MenonEun Bong LeeRemi G. ZallotEun Joo YangRenata GlowczynskaEun-jee OhRené FerreraEun-Jin ParkRene G FeichtingerEusebio ChiefariRené Klinkby StøvingEva CarroRené WenzlÉva CsőszRen-Jay SheiEva De LagoReza Akhavan-SigariEva MezeyReza Bayat MokhtariEva NeubauerRhona WinningtonEva PajkrtRicardo A BattaglinoEvaldas GirdauskasRicardo BattaglinoEvaldo FaviRicardo BoschEvangelia LiakoniRicardo BruñaEvangelia LivaniouRicardo Caballero ColladoEvangeline MantziorisRicardo Luiz Azevedo-PereiraEvangelos CholongitasRiccardo BertoloEvangelos K. KaimakamisRiccardo D'AmbrosiEvangelos PapathanasiouRiccardo Di GianfilippoEvanthia E. TripolitiRiccardo FerraciniEvelina Maria GosavRiccardo MemeoEvgenia A. GourgariRiccardo PierantoniEvidio Domingo-MusibayRiccardo SarzaniEvie MelanitouRicciarda GalandriniEvon EreifejRichard B. KruegerEwa BalcerczakRichard BerryEwa KędzierskaRichard BodnarEwa KijenskaRichard CharlewoodEwa PochećRichard E. DavisEwa RzońcaRichard E. MainsEwa StepienRichard Eric KastEwa Żurawska-PłaksejRichard G HegeleEwelina SmoktunowiczRichard H. SternsEyal SheinerRichard IJzermanEzequiel TolosaRichard KannerF. DegacheRichard MeehanFabian StöckerRichard ParrishFabio AngeliRichard RomanFabio AntonaciRichard S. FeinnFABIO FABBIANRichard W. BohannonFabio Maria TriulziRichard W. SmallingFabio PaceRickie PataniFabio PerrottaRiki ToitaFabio ZaniniRiku W. NikanderFabiola Di DatoRinaldo PellicanoFabrice MenegauxRio BoothelloFabrizia BonacinaRishein GuptaFabrizio De PontiRita PatarrãoFabrizio MatteiRitin SharmaFabrizio MontecuccoRittu HingoraniFadi NahabRitvij BowryFaheemuddin AhmedRiwa KishimotoFalk LohoffRiyaz MohamedFan FanRobert BestFan ZhangRobert D. FriedbergFarah Asa'adRobert E. AkinsFariba M. Assadi-PorterRobert FarrellFaris F BrkicRobert GabrielFarjana B. RowtherRobert HendrenFarnoosh Abbas-AghababazadehRobert HoerrFaruk HadziselimovicRobert J FontanaFauziya HassanRobert KotloskiFawaz AlzaidRobert KromerFederica FaciottiRobert LachmannFederica FogacciRobert M. BlantonFederica GaniRobert M. GaiserFederica LaguzziRobert Miranda Jr.Federica MoscucciRobert MitchellFederico BertonRobert NowakFederico FormentiRobert P. WoronieckiFederico IacopiniRobert RaffaiFederico MiglioreRobert SafirsteinFederico MussanoRobert SitarzFederico RavegliaRobert WeinbergFederico VancheriRobert WojciechFelice IasevoliRoberta AgabioFelice PetragliaRoberta BiolcatiFelice SiricoRoberta J. WardFelicetto FerraraRobert-Alain ToillonFelipe Martinez-PastorRoberto AiolfiFelix BerlthRoberto BonoFelix ClanchyRoberto CantelloFelix DreyerRoberto Dell'OmoFélix Javier Jiménez-JiménezRoberto Di PrimioFelix NickelRoberto GasparriFélix Zurita OrtegaRoberto GiovannoniFemke SlaghekkeRoberto MarciFen WangRoberto ScandurraFeng GuRoberto VitaFeng HeRobim M. RodriguesFeng Liu-SmithRobin FlynnFeng YangRobin HaringFeola MauroRobin P. Da SilvaFerdinando Antonio GulinoRobyn Laura KosinskyFerdinando MannelloRocco Salvatore CalabròFerenc IhaszRocío Martínez de PablosFereshteh PourkazemiRodica P BunaciuFergal J DuffyRodney D. BrittFernando AntoñanzasRodolfo Gomez BahamondeFernando CiveiraRodolfo IulianoFernando CordidoRodolfo MauceriFernando MazzilliRodrigo C. Fernández-ValdiviaFiammetta MonacelliRodrigo J. Carcedo GonzálezFilbert TotsinganRodrigo JiménezFiliberto Maria SeveriRodrigo LópezFilip GórskiRodrigo Martín-San AgustínFilip JansåkerRoel J.H.M. SteenbakkersFilippo CaraciRoger M KrzyżewskiFilippo M. SarulloRoger YazbeckFilippo MaselliRohan WillisFina Martínez-SolerRohit VashishtFinian MartinRohr NadjaFiona McGillicuddyRoland P. BouretteFiorella GuadagniRolf Alexander JanosiFiras KobeissyRoma PahwaFlaminia FerriRomain JouffroyFlavia NeriRomain Veyron-ChurletFlorencia PalaciosRoman ThalerFlorian KollertRomeo PatiniFlorian KurschusRonald B. BrownFlorian WegwitzRonald B. GeorgeFlorin George HorhatRonald EvansFolusakin AyoadeRonald Mauricio Sánchez-ÁvilaFor Yue TsoRonald SrokaFranca DipaolaRonald W. BerkowskyFrances ChungRonaldo Luis Da SilvaFrancesc BotetRonan MurphyFrancesc Castro-GinerRonghui LiFrancesc García GonzaloRongkung TsaiFrancesca Antonella BianchiRong-San JiangFrancesca CardarelliRongsu QiFrancesca FumagalliRory C. ReidFrancesca GattoRosa A. FabioFrancesca GhirgaRosa De StefanoFrancesca GoriniRosa María López-Pintor MuñozFrancesca IommelliRosa RojoFrancesca LavatelliRosalba SiracusaFrancesca PaoliniRosalind M. JohnFrancesca PentimalliRosalinda MadonnaFrancesca SanguedolceRosanna Di PaolaFrancesca ViazziRosanna VillaniFrancesca ZottiRosario MenéndezFrancesco AcerbiRosario SerpicoFrancesco AlbanoRosaura Esteve-PuigFrancesco AngeliniRosella TomaninFrancesco AnielloRosemary DziakFrancesco CappelliRosemary FrickerFrancesco CastagniniRosita Accardi-GheitFrancesco CenniRosita Angela CondorelliFrancesco Di LorenzoRoss MayFrancesco DiVirgilioRossana BerardiFrancesco FerrarottiRossana TrottaFrancesco LocatelliRossella AttiniFrancesco M. PacificoRossella CannarellaFrancesco MassariRossella D’AloisioFrancesco MenichettiRossella MazzilliFrancesco PanzutoRovini AmandineFrancesco PollariRoxana Maria NemeșFrancesco PrattichizzoRoxana Ramona OnofreiFrancesco SaltonRoxana StegaroiuFrancesco Saverio SorrentinoRoy Chun-Laam NgFrancesco SavinoRoy L. SoizaFrancesco SecchiRoy La ToucheFrancesco SurianoRoy MoncayoFrancesco U. S. Mattace-RasoRoy SoizaFranceso CostaRubén QueiroFranchi MarcoRüdiger MüllerFrancine DucharmeRüdiger V. SorgFrancis DumlerRudolf ArnoldFrancis P. RobertsonRudolf KorinthenbergFrancisco BorregoRudolph W. KosterFrancisco EpeldeRui M. FerreiraFrancisco GudeRui SuFrancisco Herrera-GómezRui TorresFrancisco Javier Gonzalez-BarcalaRui WangFrancisco Javier Ruiz-OjedaRuijiao XinFrancisco José González-MineroRu-Jeng TengFrancisco Miguel Martínez-ArnauRussell ConduitFrancisco Muñoz-LópezRussell HoppFrançois BerthodRussell James HoppFrancois BlachierRussell SchacharFrancois FaitotRūta VaičiūnienėFrançois PotusRuth BrauerFrank BienaiméRuth C. BorghaeiFrank BraatzRuth MilanaikFrank BühlingRuth VinallFrank FriedersdorffRuth Ya HuoFrank KroschinskyRyan FunkFrank M. DavisRyan MaysFrank PesslerRyoma MichishitaFrank StadlerRyuji HayashiFrank StahlRyujiro SasanabeFrank TackeRyusuke HoriFrank VerhoevenS.Bobo TannerFrantišek LopotSabina Antonela AntoniuFranziska EckertSabina SevcikovaFranziska PlessowSabine BartelFraser AndersonSabine GröschFred H. RodriguezSabine Hombach-KlonischFreddy SitasSabine WrengerFrédéric MarchalSabrina Di BartolomeoFrederic PamoukdjianSachiko MuraseFrederic PendinoSadaf SharfaeiFrédéric SchnellSadik A. KhuderFrederic StaermanSaeed EsmailiFrederick NaftolinSagar DuganiFrederik TrinkmannSagrario M Artín-AragónFredrik StrandSagrario Martín-AragónFrömmel ClaudiaSahar NissimFuad IraqiSaida OrtolanoFulvio D'AcquistoSaikat NandiFulvio MassaroSakamuri ReddyFulya TaylanSakthivel MuniyanFumihiko UrabeSakurai KenichiFumitaka KogaSalamah AlwahshFu-Shing LiuSalame KhalilFuyuki KametaniSalman RazviG. André NgSalvador BelloG. Kees HovinghSalvatore CarusoG. Nic RiderSalvatore PaiellaGábor GazdagSaman ModaresiGabor PozsgaiSamantha HarrisonGabriel MarcSamar KhouryGabriel SandblomSamara RiceGabriela PocoviSambasivan VenkatasubramanianGabriela S. DvekslerSameer HirjiGabriele CeccarelliSameer JainGabriele CervinoSamer JalladGabriele KollerSamuel ChangGabriele StoccoSamuel Garcia PerezGabriella BalestraSamuel O. AdegbolaGabsang LeeSamuel P. GubbelsGad SegalSamuel PeerGaetano AlfanoSamuel SeiduGaetano IsolaSamuel SilvaGaetano MarenziSamuel SnyderGaetano MottaSamuele TosattiGaetano SantulliSamy HadjadjGagan ChhabraSana DastgheybGaia FaveroSanchit AhujaGanesh SwamySandhya MatthesGang WeiSandie HaGanji Purnachandra NagarajuSandra Fátima Fernandes MartinsGanta Vijay ChaitanyaSandra FrancoGareth LemaSandra FreiGareth Morris-StiffSandra GollnickGareth R. WillisSandra Laner-PlambergerGary D. NovackSandra RugonyiGary HooperSandrine CaburetGary KohanbashSandro GelsominoGary StackSandro MatosevicGaurav GhagSandro NinniGaurav KumarSanford AuerbachGavino CasuSang Ho KwakGayatri MittalSang Hun KimGemma KaySang Wan KimGemma PratSangah ShinGenevieve HartleySangho LeeGenevieve MailhotSanjana DayalGeng-Ruei ChangSanjay AnandGen-Min LinSanjay BharatiGennaro G. BellizziSanjiv PrasharGennaro RuggieroSanna Toppila-SalmiGenoa R. WarnerSantanu BanerjeeGeoff HackettSantanu HatiGeon Ho BahnSantiago CodesidoGeorg JohnenSantiago RuizGeorge A. PapakostasSantiago Ruiz-MartínezGeorge AnifandisSara Anna BoniniGeorge BriassoulisSara ArlatiGeorge HindySara Belle DonevantGeorge J ShawSara BernardiGeorge KatselisSara Fonseca-BaezaGeorge KoliakosSara Gredmark-RussGeorge LiamisSara MocciaGeorge MaitiSara MullerGeorge NotasSara OttolenghiGeorge NoutsiosSara PalermoGeorge PapantonopoulosSara PerteghellaGeorge R. MatcukSara RezzolaGeorge W AgakSARA SULIMANGeorge W DivineSarah A. HosgoodGeorgia RagiaSarah BeckGeorgian BadicuSarah CookGeorgios D. PanosSarah HellewellGeorgios GkimpasSarah Houben-WilkeGeorgios KallifatidisSarah M. PlanchonGerald AtkinsSarah M. RabbittGerald ChiSarah MartinGerald FullerSarah NashGerald H TomkinSarah PetersonGerald VervoortSarah PringleGerd SchmitzSarah PriorGergely VargaSarah R. WellikGermaine Cornelissen-GuillaumeSarah StewartGerry CoghlanSarah VerbiestGert Jan De BorstSarang TarteyGertrude Nieuwenhuijs-MoekeSarath VijayakumarGerwin HellerSarfaraz JasdanwalaGesmi MilcovichSaša FrankGholam K. MotamediSascha KoppGiacomo CapponSatheesh ElangovanGiacomo StrapazzonSathish Kumar NatarajanGiada GiovanniniSato KeisakuGiammarco RaponiSatohiro MasudaGian Luca ColussiSatoru JoshitaGian Maria GaleazziSatoshi AkagiGiancarlo CuadraSatoshi HamadaGiancarlo NotaSatoshi HiguchiGianfranca CabidduSatoshi YanagisawaGianluca Di BellaSaturnino Marco LupiGianluca GiavaresiSaulius CicènasGianluca IacobellisSaulius LukoševičiusGianluca NazzaroSaverio GnudiGianluca PaternosterSaverio ManninoGianluca SerafiniSavino SciasciaGianluca TartagliaSavino SpadaroGianmaria D’AddazioSayali Ajit PendharkarGianna CamiciottoliSchmitt-Egenolf MarcusGideon GrossScott BrakenridgeGiedrius KanaporisScott CurryGil AwadaScott DankelGilbert DondersScott GordonGilbert SpizzoScott J. PillaGiles KirbyScott M. LangevinGillian SolesScott Martin VouriGionata FragomeniScott S. OhGiorgia BeffagnaSean C. NewtonGiorgia SpinazzolaSean CrawfordGiorgina Barbara PiccoliSean YoungGiorgina PiccoliSebastiaan Van HalGiorgio MustacchiSebastian BringsGiovanna CalabreseSebastian BürkleinGiovanna CilluffoSebastian CarballoGiovanna TrainaSebastian HahnelGiovanni C ActisSebastian KrugGiovanni CasellaSebastian MajewskiGiovanni CasiniSebastian PohlGiovanni CimminoSebastian PolakGiovanni CorsoSebastian SiebelmannGiovanni DamianiSebastian UlbertGiovanni De PergolaSebastian ZimmerGiovanni MerlinoSebastiano MassaroGiovanni MoroneSébastien AnguilleGiovanni PaolinoSecundino Cigarran GuldrisGiovanni RaffaSeddon Y. ThomasGiovanni RollaSefer ElezkurtajGiovanni SistiSeiichi KitagawaGiovanni SolitroSeiji ShiotaGiovanni TarantinoSelena TomaGiovanni ViscogliosiSelim KuçiGiovanni VitaleSelvakumar SubbianGIOVANNINO SILVESTRISenol PiskinGitte KristensenSeok Hoon JeongGiulia AbateSeonaid CleareGiulia CiccareseSeong Hye ChoiGiulia DonadelSeong-Kyun KimGiulia FisconSerafim M. OliveiraGiulia GalliSerge GauthierGiulia M. StellaSergei G. TevosianGiulio Francesco RomitiSergey FedosovGiulio IlluminatiSergey M. EfremovGiuseppe AmbrosioSergio Albiol-PérezGiuseppe Antonio MalfaSergio CoccheriGiuseppe DefeudisSergio Martinez-HervasGiuseppe GiannaccareSergio Montserrat-de La PazGiuseppe GrandeSergio RecaldeGiuseppe InsalacoSergiu PascaGiuseppe LosurdoSeth K. BechisGiuseppe LucarelliSetor K. KunutsorGiuseppe MusumeciSettimio D'AndreaGiuseppe NigriSeung AnGiuseppe PasqualettiSeung-Ki KwokGiuseppe PeraleSeungkwon YouGiuseppe PignataroŞevin TurcanGiuseppe PizzolantiSeyed Amir Danesh-SaniGiuseppe QueroSeyed Mostafa KiaGiuseppe RizzoSeyed-Ali Sadegh-ZadehGiuseppe Roberto GiammalvaShadi KatouGiuseppe SantarpinoShafiul AlamGiuseppe SimoneShailendra MauryaGiuseppe SolarinoShaina WillenGiuseppe StabileShakil AhmadGiuseppe TirinoShampa DasGiuseppe VisaniShane A. ShapiroGiuseppina BasiniShane PatmanGiuseppina PalladiniShang-Ming ZhouGladys Y.-p. KoShanika NanayakkaraGlen NowakShankar MunusamyGlenda GilliesShantanu SinghGlenis Kathleen ScaddingShao-Gang LiGloria RavegniniShaolei TengGoo-Rak KwonShao-Wen HungGopika G. NairSharad KhareGOTO YoshikazuSharad ShashwatGraham K. BarrellShariq UsmaniGrazia MaugeriSharmila FagooneeGraziella BernocchiSharon CushingGrazyna GosciniakSharon InmanGrażyna JanikowskaSharon MichelhaughGrażyna Majkowska-SkrobekSharon ReisfeldGrażyna NowickaShashvat SukhalGregory BroussardShathiyah KulandaveluGregory LewisShaun A. NguyenGregroy KatoShaun KhooGrigoris EffraimidisShawn C. RollGrueter CarrieShawn TalbotGrzegorz CieslarSheetal ParidaGrzegorz NowickiSheikh M. AlifGrzegorz WegrzynShelize KhakooGrzegorz WystrychowskiShelli R. KeslerGuanmin ChenShelly KaushikGuendalina LucariniShengda LinGuennadi SaikoSheng-Der HsuGuido GiacaloneSheng-Fan WangGuido HermeySheng-Hsiung SheuGuido IaccarinoShengmin YanGuido MollSheng-Wei FengGuido SauterShengzhi SunGuijie ChenSherard TatumGuillermo Torre-AmioneShereen EzzatGültekin TamgüneySherif BadawayGundula Gesine Schulze-TanzilSherif KhedrGunjan AroraSherry MansourGunjan Dhawan ManochaSherry StewartGunnar AntoniSherry X. YangGunnar BirgegårdShigefumi OkamotoGunther MarscheShigehisa KawakamiGuo-chang FanShigeo TakebayashiGuodong CaoShigeru ChibaGuo-Hao LinShigeru YatohGuohua LiShigetaka ShimodairaGuruswamy MaheshShih-Chun KaoGurvinder KaurShih-Pin ChenGuy CammuShih-Ping ChengGuy DecauxShih-Wei HuangGuy HansShikha SainiGuy RostokerShilpi VergheseGuy RousseauShingo IwanoGuy TrudelShingo KuwataGwendolyn P. QuinnShin-Hun JuangGwo-Tarng SheuShinichi MatsumotoGyörgy KovácsShin-ichi YokotaGytė DamulevičienėShinichiro OhshimoH HaraShinji FukuiH. Stephan GoedeeShinji MatsudaHacer AkselShinji OtaniHadi MessihaShinobu TakizawaHaesun ChoiShinsaku TogoHagir B. SulimanShinsheng YuanHak JangShinsuke MizutaniHakan PilgeShinta NishiokaHala ChaabanShintaro SukegawaHamid SuhailShinya SuzukiHamidreza AbdiShira Schecter WeinerHamza El HadiShireen KanakriHan Hsiang HuangShirin MoossaviHang XieShirley WyverHanif Javanmard KhamenehShiva KantHanjin ChoShivali S. JoshiHanna BurkhalterShivani KaushikHanna KnihtiläShivshankar ThanigaimaniHanna KomarowskaShiyu TangHanna MyśliwiecShlomi HaarHanna Przepiera-BędzakShogo HamaguchiHannah J. WeltersShogo InoueHannah OhShogo KikuchiHanne R. HaglandShogo ShinoharaHanny Al-SamkariShogo TakashibaHans DegensShokei MatsumotoHans FlaattenShona MookerjeeHans GhayeeShota NishitaniHansjörg RotheShozo YamadaHarald Kurt WidhalmShreoshi Pal ChoudhuriHarald Thune JørstadShriji N. PatelHardev PandhaShruti K. GohilHardik ModyShuangge MaHarini BejjankiShuanhu ZhouHarish SeethapathyShubhi KaushikHarneet WaliaShu-Huai TsaiHarrison W. FarberShu-Ju WuHarry AslanianShu-Ling TzengHarry MatthewsShu-Pin HuangHarsha ShanthannaShusuke HiragiHaruhiko SugimuraShuzo SatoHaruto UchinoShweta SharmaHarvey RischShyam GajavelliHassan BousbaaShyiwu WangHauke Clausen-SchaumannSiemowit MuszyńskiHawa NyendeSiew Ling TeyHayato TadaSiew-ging GongHayder Al-AubaidySiew-Kee LowHayley LewthwaiteSigal SviriHe LiSigne Abitz WintherHeather E. DanyshSigrid Bjerge GribsholtHeather F. SmithSigrun EickHeather Jean MedburySijan BasnetHeather WalkerSilva Katuŝić HećimovićHector S IzurietaSilvana GalderisiHedwin Kitdorlang DkharSilvia AlemanyHeidi HongistoSilvia BistiHeidi NeubauerSilvia CorrochanoHeidrun LewaldSilvia GalliHeike WeighardtSilvia GaragnaHeiko GrafSilvia LaiHeinrich GerdingSilvia Laura BoselloHeinz BaumannSilvia MongodiHeinz LohrerSilvia MonticoneHeinz WinklerSilvia NovíoHelal HettaSilvio AntoniakHelder AndreSilviu AlbuHelen BranthwaiteSim K. SinghraoHelen EdwardsSimon AllenHelen FillmoreSimon AllisonHelen HuSimon BodyHelen L. CarlsonSimon BowlerHelen M. CrohnSimon CooperHelen McGuireSimon Daniel Israeli-KornHelena LingeSimon FoxHelmut H. PopperSimon PodnarHelmut HopferSimon VautierHema KothariSimon W. RabkinHemant GiriSimona LaurinoHemant PanditSimona OrtelliHemanth KandikattuSimona RoateşiHenk Van BeuningenSimone AgostiniHenning BliddalSimone BersiniHenri AarnivalaSimone ReppermundHenriette LernerSimone RiberoHenrik FalhammarSimone RossiHenrike HeyneSimone VidaleHenri-Pierre LassalleSinead AhernHenrique AlmeidaSinead M. SmithHenry A. LeopoldSing-Chung LiHenry LukaskiSinisa MarkovicHeon Yung GeeSinthia Bosnic-AnticevichHerbert Michael HeiseSiobhan MorseHerbert RiechelmannSirwan M. HadadHerbert StanglSissel MoltuHermann FrieboesSiv FonnesHermann WriggeSivaraj Mohana SundaramHermien HartogSivaraman PrakasamHershel RaffSixto Garcia-CastineirasHervé ReychlerSjors VerlaanHerwig GerlachSkaidrius MiliauskasHetian LeiSlaven PikijaHeung Kyu LeeSlavena VylkovaHeyu NiSlawomir GonkowskiHicham AjamiSmadar Valérie TourjmanHideaki KawabataSmitha SripathyHideaki OkajimaSmriti KrishnaHideaki SoyaSofia AvnetHidekazu SuzukiSofia El Manouni El HassaniHideki MiyachiSofia KottouHideki MoriSofia TheodoropoulouHidenori TakahashiSohail Abdul SalimHideo NakanishiSohee JeonHidetada HirakawaSohsuke YamadaHideyuki KawauchiSolange LandrevilleHilary A. KennySolène M. EvrardHimali SomaweeraSoliman KhatibHinanit KoltaiSolomon MenahemHira Lal GoelSomashekar G. KrishnaHiraku OnoSomchai AmornyotinHirayuki EnomotoSomnath SinghHiroaki MizukamiSompop BencharitHiroaki NakaSong LiHiroaki NakagawaSonia Do CarmoHiroaki TanakaSonia ScarfìHiroetsu SuzukiSonia ShirinHirofumi HiokiSónia SousaHiroki SasakiSonia ValletHiromichi YumotoSontake VishwarajHironao HozumiSookyung LeeHirono IriuchishimaSoon Kil KwonHiroshi KurokiSophie DuportHiroshi NishiokaSophie LiabeufHiroshi TanakaSören MöllerHiroshi YokomichiSorin HostiucHiroshi YoshidaSorin MihaliHirotaka OnishiSoroku YagihashiHirotaka SugaSotiria PaliouraHirotaka TashiroSowmya ViswanathanHiroteru KamimuraSpencer P. LakeHirotsugu HashimotoSperanza RubattuHirotsugu OhkuboSpyridon P. DourakisHiroyuki IshiyamaSpyridoula MarakaHiroyuki KatohSrba MijailovichHiroyuki KinoshitaSreeharsha GurrapuHiroyuki NoderaSreenath NairHiroyuki ShimadaSreepriya PramodHiroyuki TodaSridhar ManiHiroyuki TomitaSrikanth BellaryHiroyuki UmegakiSrikrishnan MallipeddiHiroyuki YoshiharaSripathi M. SurebanHisashi KogaSri-Rajasekhar KothapalliHisashi TakeuchiSriram GubbiHitoshi ShitaraSruthi RavindranathanHo Lam TangStaci MartinHo-Chang JeongStalhammar GustavHojong ChoiStavros J. BaloyannisHolger A. RamboldStavroula BaritakiHollis O'NealSteen J. MadsenHolly LeddyStefan HaemmigHong ChangStefan HohausHong MaStefan KluzekHong S. LuStefan KreyerHong Soon-CheolStefan MühlebachHong YuStefan MutterHong-Bo ZhaoStefan NaydenovHong-Jin LeeStefan NierkensHongseog SeoStefan NilssonHongyi ZhouStefan PetersHorace MassaStefan TanglHorng Ruey ChuaStefan WelterHortensia De La FuenteStefan WoelflHosim SohStefania MarcuzzoHossein GholizadehStefania ZanussiHou-Chuan LaiStefano CarossaHouria DaimiStefano De ServiHoward DonningerStefano GaspariniHsiao-Yean ChiuStefano ManagòHsin-Fang ChungStefano MenzoHsu-Wei FangStefano PaganoHuabiao ChenStefano PiccaHuang-Ping YuStefano RapiHuaping ChenStefano RavaioliHubert MarotteStefanos BonovasHubert ScharnaglStefany PrimeauxHuey-Chun HuangStéphane J. C. ManciniHugo De BruynStephane RetyHugo FernandesStephanie E. HallHugo FragaStephanie FilleurHugo KatusStephanie G.C. KroezeHuhn KarenStephanie K. DouganHui Jai LeeStephanie StanfordHui LyuStephen A. GlazerHui-Chun HuangStephen CornishHui-hua ChangStephen E GitelmanHumayun IrshadStephen HumphriesHung SunStephen L. BallHung-Pin TuStephen L. JonesHuub H. Van RossumStephen MakinHyang Woon LeeStephen MullinHyanghee KimStephen N. WaggonerHye One KimStephen R. PlymateHyeon JeongStephen RayportHylke J.F. BrenkmanStephen T McSorleyHyoung-Gook KimStephen W. TuffsHyun SeokStevan NikolinHyung-Jun KimSteve BainHyungseok JangSteve EllisIan A. MylesSteve FeldmanIan L.P. BealesSteve HyerIben AxénSteve ShnyderIbrahim El-BattrawySteven BrodyIchiro OkanoSteven E. MutsaersIddo Z. Ben-DovSteven G. KovenIevgen KoliesnikSteven HartIfy R. MordiSteven L. WarsofIgnacio GiménezSteven LevyIgnacio Novo-VeleiroSteven O'ReillyIgor A. ZolotukhinSteven PapachristouIgor BadoStewart AlbertIgor GrabovacStuti MisraIgor LoniewskiStygar DominikaIgor ŁoniewskiStylianos RavanidisIkenna C. EzeSu Jung Oh-HohenhorstIk-Hyun YounSu SongIlaria RoatoSubash HeraganahallyIlayaraja MuthuramuSubbaramiah SridharIldebrando AppollonioSubhradip MukhopadhyayIldem AkermanSubhransu SahooIldiko RaczSudheer RavuriIleana BaldiSuguru YamamotoIlia BeberashviliSu-Hyun ShimIlias ElmoukiSu-Jin HeoIlja L KruglikovSumeet SolankiIlona CsizmadiSumit DasIlse DaehnSumit MukherjeeIlse RoomanSun Hee DoIlze KonradeSuneel KumarIma DovinovaSung Kweon ChoImam Mohammad ZafarSung-Hsin KuoIman MomkenSunil KumarImo HoeferSunil PauwaaImran H. KhanSunil SwamiImran HouseSun-Young LeeImran SiddiquiSuowen XuIn Sik YunSupreet KhanalIna BeintnerSupriti SamantarayInaam NakchbandiSuraj BhatIñaki MiltonSurbhi ChamariaIndrajit ChowdhurySurendra DasariIneke van der HamSuresh SamsonInes Bilic-ĆurčićSuresh VeeramaniInes MármolSusan GoodmanInga PeterSusan HillerInga VogesSusan J. WenzeInge TetensSusan M NeufeldInger AhlstrandSusan Philippa WalkerIngerid ArboSusana BalcellsInhae KangSusanna A. CurtisIñigo LandaSusanna FortunatoIn-Jae OhSusanne GrubeInna P. GladyshevaSusanne KarbachIoana IonSusanne MohrIoana MozosSusanne QuallichIoana R. PrestonSushama SivakumarIoanna PettaSusheel Kumar GunasekarIoannis BakoyiannisSusheela HadigalIoannis MavridisSushilkumar Satish GuptaIoannis MavroudisSusumu OokawaraIoannis VasileiadisSuvi KuivanenIoannis VogiatzisSuzanne RussoIon CristóbalSuzanne VerlhacIona NovakSuzie FerrieIona SchusterSveinung Wergeland SorbyeIori KisuSven SaussezIppokratis MessaritakisSven VetterIra HoferSvenja SydorIraide AllozaSverre E. KjeldsenIrena GlazarSvetlana CherlinIrena ManovSvetlana KurklinskyIrene DogliottiSwapnil BawageIrene HarrisSwapnil ShewaleIris E. EderSwastik PhuleraIris H. JonkersSy Duong-QuyIrun BhanSylvain BourgoinIsaac Jonathan PomeraniecSylvain GrenierIsabel M. McFarlaneSylvia KirchengastIsabel Rodríguez GarcíaSylvie BabajkoIsabel SevillaSyn-Heyog ImIsabell WitzelSytze De RoockIsabella PanfoliT. Jeroen N. HiltermannIsabella ZanellaT. Y. Alvin LiuIsabelle ArnetT.S. (Tjip) Van Der WerfIsabelle Gouin-ThibaultTaco GosensIsabelle HoorensTadahiko IshiyamaIsao OtsukaTadahiro ShojiIsao ShiraishiTadashi SofueIsha SharmaTadayoshi KagiyaIsmaeel YunusaTadayuki OshimaIsmael SeáñezTadeusz OsadnikIsmail H. Al-AbdullahTae Hyun KimIssac SachmechiTag Keun YooIssam TanoubiTahsin Stefan BarakatIstvan KovaneczTaichi IkedoI-Te LeeTaina CardosoItziar Abete GoñiTaisun KimItziar AlkortaTak Pan WongIulia-Cristina BagiuTakaaki HasegawaIulia-Diana MuraruTakahide KagawaIuliia PolinaTakahiro InoueIvan KruljacTakahiro OkumuraIvan KushkevychTakahiro YasuiIvan PrestaTakakuni MakiIvana TrapaniTakanori EguchiIvar A. EideTakao ImaiIwona Krela-KaźmierczakTakashi AkamizuIzabela ZakrockaTakashi NakamotoJ. Kim RossTakashi YazawaJ. Steven HataTakashige AbeJacek MalejczykTakatsugu OkegawaJacek NasiłowskiTakayuki IshiharaJacek SzeligaTakayuki MasakiJack L TurbanTakekazu MiyoshiJacky Y. SuenTakenori InomataJacob BabuTakeru NabetaJacob FreedmanTakeshi KeraJacob JosephTakeshi KitaiJacob MeyTakuji IwashitaJacopo SabbatinelliTakuma ShibataJacqueline HoTakumi AbeJacqueline JozaTamás Kovács-ÖllerJacquelyn D. Wiersma-MosleyTamas SzakmanyJacques GalipeauTanima BoseJacques PirenneTanis FentonJacques RobertTanja Grubić KezeleJacques ZimmerTao-Hsin TungJade LoggerTara PerrotJadranka A. SpahijaTara SigdelJae Chan KimTarek AlsaiedJae Ill ShinTarek NaguibJaegyun OhTarik SammourJaideep DhariwalTariq MalikJaime Butler-DawsonTaro HorinoJaime EstebanTaroh SatohJaime SanchoTatiana SimaoJakub ChwastekTatiana V. KudryashovaJakub TaradajTatijana ZemunikJames BerkleyTatsuo OkuiJames BluettTatsuo ShimuraJames BrasicTatsuya YoshiharaJames D KeanTawnya Wilson PastuckJames D. MancusoTe-Huei YehJames H. LewisTelmo PereiraJames HorowitzTeodora Alexa-StratulatJames J. GoedertTerence A. ImberyJames KruegerTeresa Bernadette SteinbichlerJames LangTeresa GaglianoJames MainprizeTeresa M. LeeJames MelroseTeresa Sosa DíazJames N. SleighTerrence DonohueJames P. CorsettiTerry McMorrisJames P. HardwickTerry W. MoodyJames R. CouchTeruaki NakatsujiJames R. EckmanTeruhiko ImamuraJames RobinsonTeruyuki MatsuokaJames S. PowersTessa LordJames SmythTessa PeasgoodJames StaffordTessel GaleslootJames TsaiTetsuo NishikawaJames W GeddesTetsuro KobayashiJames WilsonTetsuya HommaJameson ForsterTetsuya TeradaJan BilskiTetsuya YumotoJan BrozThaddeus BirchardJan ChojnackiThamarai SchneidersJan D. SchmittoThanh D. HoangJan GunstTharindumala AbeywardanaJan H RosenvingeTheis Skovsgaard ItenovJan HagemannTheodora BrisimiJan HomolakTheodore KarapanayiotidesJan KehrmannTheodoros AslanidisJan KrizTheodoros EleftheriadisJan KrumsiekTheodoros TokasJan NovákTheofilos PapadopoulosJan PlutaTheresa HautzJan RossaintTheresa L. WhitesideJan RuffieuxTheresa M BuschJan SimoniTheresa MadalineJan SkodaThibault GallavardinJana KatuchovaThierry MorineauJane AntonyThimios A. MitsiadisJane ChudleighThiruvengadam MunirajJane FletcherThomas A ZikosJane LiesveldThomas A. AgbaedengJane LittleThomas AndlJanet A. FooteThomas BrettJanet LighthouseThomas BroganJanet RichmondThomas BschleipferJanett BarbareskoThomas D. CoatesJan-Henning KlusmannThomas DaubonJanis KlovinsThomas E. MürdterJanna G. KoppeThomas F.J. KingJanne VeerbeekThomas G. PoderJanosch HellerThomas HorvatitsJanusz BlasiakThomas J GuttusoJanusz SkrzypeckiThomas JanssensJared BarrottThomas JonesJari Antero LaukkanenThomas LambertJaromir GumulecThomas LaneJarosław DomaradzkiThomas MastJarosław J. SakThomas MawhinneyJaroslaw KasprzakThomas McdonnellJaroslaw PiskorskiThomas MeinelJasbir UpadhyayaThomas P JohnstonJasmin ArrichThomas PabstJasmin GrischkeThomas Richard NiethammerJason BlackardThomas Scot MurrayJason ChengThomas SkripuletzJason CollierThomas VolkenJason D. SimmonsThor UelandJason L. JohnsonThorsten KirschJason MockThorsten R. DöppnerJason ZastreThorsten SchinkeJasper MostTiago GranjaJatinder LambaTian TianJaume Coll-FontTianfu WuJavier A. NeyraTiffany GillJavier Alvaro-Afonso FranciscoTiffany GrahamJavier Ata-AliTiffany JonesJavier CejudoTigran PoghosyanJavier De Las HerasTill Jasper MeyerJavier Donate-CorreaTillmann H.C. KrugerJavier Garcia-PardoTilman WolterJavier GilTilo NiemannJavier HerreroTim Hideaki TanakaJavier Molina-CerrilloTim SciallaJavier Ochoa-ReparazTimm H. WesthoffJavier OrtegaTimo GnambsJavier P. GisbertTimo GreinerJa-Won KooTimo RathJaw-Yuan WangTimon VandammeJay IrwinTimothy Bryan Franci WoodfieldJay PooleTimothy HuangJay S. MishraTimothy PaderaJay W. MasonTimothy RittmanJaya AseervathamTimothy SlyJaya GnanaprakasamTina Tičinović KurirJayarama GunajeTineke E. BuffartJaydeep BhatTinen IlesJaydira Del RiveroTing Yuan David ChengJayson StoffmanTino ZähleJean A. BallwegTJ MurtolaJean A. BernatchezTjitske HielkemaJean BertheletTobias BäumerJean Christophe GrisTobias BruegmannJean E. AaronTobias DallengaJean M. ConnorsTobias KramerJean Philippe Drouin-ChartierTobias NijmanJean-Emmanuel BibaultTobias PiegelerJean-François AugustoToby CandlerJean-François ChenotTodd FoxJeanie TryggestadTodd MargolisJeanine M. BuchanichToh Leong TanJeanine Van KlinkTom SnellingJean-louis VincentTomas DuffyJean-Marc LobaccaroTomás Gallego-IzquierdoJeannet LauenborgTomas PoskusJean-paul ColletTomasz BondaJean-Paul DecuypereTomasz BrudekJean-Pierre ZellwegerTomasz GosiewskiJean-Yves ScoazecTomasz KantykaJee Hwan KimTomasz KostkaJeevisha BajajTomasz PowrózekJeeyun AhnTomasz RakowskiJeffrey A. KrauTomasz ŚledzińskiJeffrey GimbleTomasz ZapolskiJeffrey L. AndersonTomer M. MarkJeffrey McCulloughTommaso AngeloneJeffrey NortonTommaso FelicettiJeffrey R. AndolinaTommaso GoriJehan El-JawhariTommi VatanenJelle De WitTommy LiJelle VehofTomoaki IshigamiJelmer AlsmaTomoaki MatsumoriJemima AlbaydaTomoaki MoriokaJen-Chang YangTomoaki TakataJeng-Fan LoTomohiro BitoJeng-Ren DuannTomohiro OsakiJennica L. ZaroTomomi FujiiJennifer BarkinTomoo KishabaJennifer BrunoTomoya YokotaJennifer CoriTomoyuki KawamuraJennifer EtnierTomoyuki TakuraJennifer HaynesToni Celia-TerrassaJennifer HulingToni T. SeppäläJennifer J. CrowleyTonino TrainiJennifer L MulliganTony BadrickJennifer L. HartwellTony CassidyJennifer M. GrossmanTor Henrik TvedtJennifer MarinoTorben Riis RasmussenJennifer MunkleyToru IshiharaJennifer N. PhanToru IwasaJennifer PattersonToru KotaniJennifer R. MammenToru YagoJennifer S. GravesToshi NagataJennifer S. LeesToshiaki OharaJennifer SimkinToshihiro InoueJenny DrottToshihiro NishizawaJenny EkbergToshihiro Sakurai SakuraiJens SpiesshoeferToshihiro TsurudaJens-Ulrik JensenToshimitsu YamaokaJérémie GautheronToshinari MinamotoJeremy Jian Wei ChenToshio KuboJeremy LagrangeToshiyasu AmanoJeremy R. StoddartToshiyuki UCHIDAJeremy SokoloveTosifa MemonJer-Ming ChangTracy ReibelJeroen De JongeTravis E. GrotzJeroen L.A. van VugtTrevor StewardJerome CanadyTrevor ThompsonJérôme LaurinTrish BergerJerome NigouTse-Hung HuangJerry T. DangTsering StobdanJerry W. SimeckaTsuneaki KenzakaJerzy NarlochTsung-Chien LuJerzy P. SzaflarskiTsung-Hsuan LaiJerzy-Roch NoferTsung-I HsuJesse N. MillsTsung-Jen WangJessica Amber JenningsTsuruya KazuhikoJessica BlackburnTsutomu SakuradaJessica Dal ColTsu-Yao ChengJessica L. FletcherTsuyoshi TagawaJessica M. CottreauTsvetelina VelikovaJessie M. SutherlandTsvetoslav GeorgievJesús ArenasTudor Lucian PopJesús DevesaTuija LeskinenJesús EspadaTunde CsepanyJesús F. Bermejo-MartinTze MokJetan H. BadhiwalaTzeon-Jye ChiouJia WenTze-Sian ChanJia-Feng ChangTzu-Fei WangJian XuTzuwei WangJianfeng LiuUgo CavallaroJianying ZhangUgo TestaJianzhong HuUjjwal RamtekkarJiaqing HaoUlla RomildJi-Dung LuoUlla SovioJie (Jane) WangUlrich Friedrich WellnerJie GaoUlrich JuliusJie ZhuUlrich KnappeJi-Hyun JangUlrich PecksJijun HuangUlrika MarklundJill M. PlevinskyUlrika Snygg-MartinJill SuttonUlrike RESCHJill WaalenUma GautamJillian Vinall MillerUmberto MalapelleJi-Man ParkUmberto SiméoniJimi FrancisUmberto TarantinoJin H. HanUmile Giuseppe LongoJin LiUrsula FearonJin Won HuhUrsula Loizides-MangoldJinfeng ZhaoUrszula CytlakJing FengUrvish PatelJing PanUte PanzenboeckJing PangUthayashanker EzekielJingchuan GuoVáclav RancJin-Ming WuVadim KlimontovJinthe Van LoenhoutVaidotas MarozasJin-Wook KimValentin BenzingJitendra Kumar TripathiValentina De FalcoJiunn-Horng KangValentina LiakinaJi-won RyuValentina MassaJixiang ZhangValentina SalaJoachim KunzValentina SancisiJoan Torras-AmbròsValentine BrousseJoana F. B. BarataValérie LecureurJoana GameiroValerie LeeJoanna Achinger-KaweckaValerio D'OraziJoanna BojarskaValerio GallottaJoanna DidkowskaVallì De ReJoanna Gdula-ArgasinskaVanathi PerumalJoanna Matowicka-KarnaVance ZemonJoanna MazurVanessa DesantisJoanna RossowskaVanessa MateusJoanna Saluk-BijakVanessa Meier-StephensonJoanna WietrzykVanessa NomelliniJoanne N. Nyarangi-DixVanina MeyssonnierJoanne TanVannessa LeungJoão M. MirandaVarga János TamásJoao Miguel SantosVarvara TrachanaJoão Nobre CardosoVarykina G. ThackrayJoão RRamalho-SantosVasanth KumarJoaquín TimonedaVasilios AthyrosJoar GuterstamVasilios BerdoukasJochem M JansenVasilios E PapaioannouJochen KleinVasily KashtalapJochen MutschlerVatsalya VatsalyaJody YeVeeranna MaddipatiJoe Leign SimpsonVelga SudrabaJoe VergheseVenerino PolettiJoël BelminVenkateswara Reddy GogulamudiJoel NeugartenVenu Gopal PillarisettyJoel W. HayVera A. PaulsonJoey A ContrerasVera CappellettiJogender MehlaVered RazJohan EngdahlVerónica Barroso-GarcíaJohan HambraeusVeronica DusiJohan WestinVeronika HuntosovaJohann SellnerVeronika MyasoedovaJohanna A KnipperVeronique GingrasJohanna Olson-KennedyVéronique KemmelJohanna WestraVesna D. GarovicJohannes BreyerVesta SteiblieneJohannes ClausenVibhash SharmaJohannes GreinerVicenç FalcóJohannes HausmannVicente BodiJohannes Hendrikus SmitVicente Calixto Zanon-MorenoJohannes OttVictor C. KokJohannes Van Den AnkerVictor ChedidJohannes WinklerVictor E. A. GerdesJohn A. PetrosVictor J. VerwaalJohn C. HaydenVictor MayJohn D. HaleyVictor VictorJohn D. KakisisVictoria M. LeavittJohn E.A. BlairVictoria WorkmanJohn FlackVidhya RaoJohn FunderVidmantas VaičiulisJohn HopperVijay RamakrishnanJohn J. NigroVijay RamaniJohn K TriantafillidisVijayabhaskar VeeravalliJohn K. YueVijayalaxmi GuptaJohn KonhilasVijaykumar SutariyaJohn M. LachinVikrant RaiJohn PandolfinoVilma DženkevičiūtėJohn ParkVincent De ConinckJohn PasiVincent DochezJohn Paul OliveriaVincent LavouéJohn S. TregoningVincent LeeJohn T. BatesVincent SegersJohn TanVincent VendittoJohn TeiberVincent ZimmerJohn UmVincenzo BrunoJohn W. FarrellVincenzo Davide PalumboJohn Wayland Farrell IIIVincenzo De FilippisJohnathon D. AndersonVincenzo GuidettiJolanda KluinVincenzo LionettiJolanta Klukowska-RötzlerVincenzo MatteiJolanta Patro-MałyszaVincenzo PotaJolanta WeaverVincenzo SavarinoJon B. SuzukiVineet AgrawalJon IrazustaVini NagarajJon Jarløv RasmussenVirendra ChaudhriJon RussellVirginia D. SteenJonas KolbenschlagVirginia PalegJonas P BecktorVishwanath SankarasubramanianJonas VerbruggheVita GolubovskayaJonathan BroadbentVitaliano Francesco MuziiJonathan Dale CaseyVito PavoneJonathan G HardmanVito RacanelliJonathan H. SchatzVittorio PiraniJonathan KopechekVittorio SimeonJonathan KopelVivian De WaardJonathan LovellViviana MeravigliaJonathan PlasenciaVladimir LazarevićJonathan R SkinnerVladimir SaenkoJonathan RhodesVolker M. LauschkeJonathan S. BoomerVytautė PečiulienėJone A. StanleyWadih MaaloufJong Hyuk SungWael Al ZoughbiJong Yun LeeWai-Ching LamJong-Shyan WangWai-Lung NgJong-woo ChoiWalid Mahmoud KhaliliaJoo Hyoung LeeWalter L. StrohmaierJordan YaronWalter ReinhardtJordi BoverWan Mohd Azam Wan Mohd YunusJordi Gracia-SanchoWangdo KimJordi MolgoWan-Jung LuJörg HuberWankun XieJorge Arturo Santos LópezWarwick ButtJorge CervantesWasim S. KhanJorge H VillafañeWayne RobertsJorge Hugo VillafañeWayne W. GrodyJorge L GamboaWei LeiJorge LascanoWeicai ZhangJorge OrtizWei-Chieh LeeJorge PerazaWei-Chyou WeiJorma A PaavonenWeijia ZhangJos BloemersWeijie ZhangJosanne VassalloWeijing SunJosé A. AguirreWeijuan LiJose A. CostoyaWeiling YuJosé Antonio García VidalWeiqin ChenJose Antonio Gonzalez-JuradoWei-Ting ChaoJose Antonio Vega AlvarezWen LinJosé BragançaWen-Cheng ChenJose CalbetWen-Chi ChouJosé Carlos Fernández-GarcíaWenchun ChenJosé Eduardo Maté-Sánchez de ValWen-Chung HuangJosé Gutiérrez MaldonadoWen-Chung TsaiJose L. Flores-GuerreroWen-Fu LaiJose L. Gómez-UrquizaWen-Kai WengJosé Luis Calvo GuiradoWen-Yi YangJosé Luis Calvo-GuiradoWenyu ZhangJosé M. MatésWerner StenzelJose M. MestreWerner ZimmerliJosé Manuel Ferreira MachadoWesley HayesJosé Manuel Moreno VillaresWilfrid JänigJOSE MANUEL MORENO-VILLARESWilfried A. KuesJosé María AyusoWilfried GyselaersJosé MartinhoWilhelm MistiaenJosé Martín-NietoWilliam BobierJose Medina-InojosaWilliam C. (Trey) PutnamJosé Miguel UrraWilliam F. Crowley Jr.Jose MoranWilliam GrantJose Sanchez-AlcazarWilliam HallJose ThekkiniathWilliam L WhittierJosef FallerWilliam McCownJosef FinestererWilliam ParkerJosefin AhnströmWilliam R. ReedJoseph A. CarcilloWilliam RomineJoseph AtallahWilliam T. HuJoseph F. PierreWilliam ThomasJoseph L. DemerWilliam WangJoseph M. PierreWimal PathmasiriJoseph MarinoWinfred WangJoseph RispoliWinston LiauwJoseph SalisburyWisit CheungpasitpornJoseph TaubeWladyslaw JanuszewiczJoseph W. GordonWojciech JelskiJosephine RidleyWojciech RozekJoshua BarzilayWolfgang BaehrJoshua D. BrownWolfgang FischbachJoshua J. ArmstrongWolfgang H. SommerJoshua M. HareWolfgang RottbauerJosip A. BorovacWon Ho KimJosko BozicWon-sik HamJoško MarkićWoon-Man KungJouke DijkstraWouter KokJouni LauronenWujin SunJoyce ThompsonXabier UrraJózsef SzalmaXavier MoissetJr-Jiun LiouXi ChenJuan Carlos De VicenteXi ChengJuan Felipe LucenaXiang XueJuan I. Pérez-CalvoXiaobo ZhouJuan J. BadimonXiaodong WangJuan José SantamaríaXiaohu HuangJuan Luis Rodríguez HermosaXiaojun DuJuan M. MachimbarrenaXiaoli TangJuan MielgoXiaoming ZhangJuan P RodrigoXiaopeng JiJuan P. MartínezXiaoxiao LiuJuan P. RodrigoXiaoying CuiJuan Pablo De Rivero VaccariXin GuoJuan QinXin XuJuan R TejedoXincheng YaoJuan Ramón GimenoXingcheng ChenJuan ZhouXingjuan ChenJuana GallarXinli DuJuanita HodaxXinyin JiangJuan-Jose SeguraXIONGHAO LINJubi E.de HaanXiyong LiuJu-Chien ChengXosé Antón Vila SobrinoJudith CrowellXuejun WangJudith L. LuborskyXuezhi JiangJudith MontagYacov FogelmanJudith SluimerYa-Fang MeiJudy KarpYagna JarajapuJuergen HoneggerYago Leira FeijóoJuha PalonevaYan LiangJuhani AiraksinenYan LiuJu-Hyoung LeeYanbo YuJules C. HancoxYandong LaiJulia CostaYang ChenJulia FuchsYang LongJulia MackYang PengJulia RiedlYang ShiJulia SchumannYang ZhangJulia SteinhilberYannick SaalbergJulian KünzelYanqin RenJuliana De Freitas GermanoYan-Shen ShanJuliana Imgenberg-KreuzYao - Lung ChangJuliana MarottiYao-Chi ChuangJulie ChoisneYara HoseinJulie DutilYasuaki NakajimaJulie LangYasuaki TokuhashiJulie PrescottYasuhiko MatsumotoJulie Reisz HainesYasuhiro TakahashiJulie Y. JiYasukazu YonetaniJulien EdelineYasunori OtowaJulien FreitagYasuomi OuchiJulien GuiotYasushi ShibataJulien HadouxYasushige ShinguJulien HoganYasuyuki FukuharaJulien Saint-PolYat-Ching TongJulien Van GrevenyngheYehuda ShienfeldJulijan KabiljoYen-Han LeeJulio Francisco Jiménez-BonillaYen-Ko LinJulio MoralesYen-Kuang YangJulio PascualYeong-Ren ChenJulita KulbackaYibin XieJun HuaYicheng NiJun LiuYi-Chiung HsuJun NakaeYifat ManorJun OhYiguang LinJun ZhangYi-Hong ChouJunaid RafiYih-Sharng ChenJunchao TongYi-Hung ChiangJung Han KimYih-Yuan ChenJung Hong HaYi-Jang LeeJung-Hee RyuYi-Jen LiaoJungHwan OhYijie WangJunghyun KimYilang TangJungil ChoiYi-Ling YeJung-woo ChaeYinan HuJungwoo LeeYinchen SongJunhui HuYing LiJun-ichi HanaiYing-Hung LinJun-ichi KawadaYing-Jie PengJunichiro IrieYi-Ting HsiehJun-ichiro KogaYiwen LiJun-Il YooYixing DuJunji TakayaYoav BrozaJunji UchidaYoav YinonJunjiang SunYohann DabiJunjie ZhuYohannes Woubishet WoldeamanuelJun-Jun YehYohei DoiJunko TanakaYohei HisadaJunko YoshidaYohei MineharuJun-Te HsuYohei MiyamotoJürg NüeschYoichi ImaiJurriaan B. TuynmanYoichiro YoshidaJussi PihlajamäkiYoji OguraJustin KreuterYoji TakeuchiJustin LeesYon-Cheong WongJustin Maximilian MittelstaedtYong Chan AhnJustin P. Van BeusecumYong Jin KimJustin RichnerYong WangJustine DebeliusYong-Chen LuJustyna WidomskaYonggang PeiJuzar AliYonggeun HongJyh-Chin YangYonggoo KimJyh-fei LiaoYong-Ho ParkK. JoostenYongjae YooKabilan VelliyagounderYonglong WeiKabirullah LutfyYong-mi KimKadir OzaltinYoon Jeong ChoiKagaku AzumaYori GidronKalyan Nagulapalli VenkataYoshiaki SatoKamal JoshiYoshifumi ItohKamayani SinghYoshihiko KakinumaKang-Ho ChoiYoshihiko KannoKang-Ming ChangYoshihiko TachiKannan ThanikachalamYoshihiko YanoKareen HeinzeYoshihisa MatsukawaKarel AllegaertYoshiro HoraiKarel RoubikYoshitaka ToyomasuKaren BoazYoshiyasu UchiyamaKaren LindsayYosky KataokaKarim D MahmoudYosuke NakataniKarim HamaouiYounes SmaniKarin BoomarsYoung Jin LeeKarin DiserensYoung Joo ShinKarin KosulinYoung Whan ChoiKarin NylanderYoungjae YouKarina GaloianYoung-Ji ShiaoKarine PinelYoung-Jun LimKarl DrlicaYouqiang KeKarl KingsleyYousif SubhiKarl KoschutnigYoussef HibaouiKarl KuchlerYoussra Al-HilalyKarl LechtreckYu KoyamaKarline Wilson-MitchellYu LiuKarn WijarnpreechaYu ZhuKarol KamińskiYuan HuKarol SestakYuanjie PangKarolina Elizabeth SzummerYuan-Tzu LanKarolina GrątYuanyuan DuanKaroline Mayer-PickelYuanYuan WangKarsten MünstedtYu-Chen LeeKarsten SchrörYu-Ching WenKarsten ZenglerYue ShengKarthik TappaYue WangKasi Viswanatharaju RuddrarajuYueh-Ying HanKasia KozlowskaYuh BabaKasper StokbroYuhi FujimotoKatarzyna A. PirógYuichi MaedaKatarzyna GórskaYuji KamijoKatarzyna GurzawskaYuji MurakawaKatarzyna HooksYuji ShimizuKatarzyna Komosinska-VassevYuji UchioKatarzyna KotfisYuki FujitaKatarzyna Kozar-KaminskaYukihide MaedaKatarzyna M. LisowskaYukinori TakenakaKatarzyna MichalekYukio KageyamaKatarzyna NeubauerYukio MaruyamaKatarzyna NowinskaYukio YuzawaKatarzyna NowomiejskaYuko OnoKatarzyna Skórzyńska-DziduszkoYula SerpanosKatarzyna SocałaYun Jung BaeKate BramhamYun Kyung KimKate HimmelmannYunfeng LiuKaterina KavalidouYung-Chih ChenKaterina ProkopiouYung-Kai HuangKatherine A. KleinYunli ZhouKatherine AlpaughYuri BattagliaKatherine BaldockYury KhelemskyKatherine E. StraffordYusuke KuroseKatherine J. MotylYusuke SakaguchiKatherine SchaumbergYusuke SasabuchiKathleen Boesze-BattagliaYusuke ShimakawaKathleen DenisYusuke TaniyamaKathleen GillespieYusuke TomitaKathleen HamYusuke YamamotoKathleen K. ChristiansYutaka ItokazuKathleen M. O’NeilYutaka KishidaKathrin BeckerYutaka MifuneKathrin Eve LewandowskiYutaka SeinoKathrin SchagYuto TanakaKathryn Eby BeckermannYuval RamotKathryn StowellYuyan HanKathryn V. DalrympleYuzaiful Md YusofKathy BoutisYves BorbélyKatia MareschiYves JammesKatie DruceYves MenezoKatrin RauenYvonne A. DanielKatrina SchmidYvonne Jane SummersKatrina TongaZachary A. VesoulisKatsumi HigakiZachary EvansKatsuyuki TanabeZachary R. SmithKaty De Paco MatallanaZachary WatsonKaushik MukherjeeZachary Z. FreybergKaveh RoshanbinfarZack OakeyKavitha YaddanapudiZaferis LouvarisKazuaki TokodaiZahra JalalKazuhide WatanabeZahra MalekiKazuhiko NakayamaZaid AbassiKazuhiko TakeuchiZaid Al-QurayshiKazuhiko YanaiZaid H. MaayahKazuhiro IkedaZakir UddinKazuhiro KatadaŻaneta Kimber-TrojnarKazuhiro KawamuraZaneta M. ThayerKazuhiro OgaiZbigniew JabłonowskiKazuhiro P. IzawaZeeshan FazalKazumi KawataZeynep Alpay SavasanKazunori ShimomuraZeynep ErogluKazuo YudohZhanping LuKazutaka NanbaZhanqi ZhaoKazuyoshi IshidaZhanxiang WangKedra WallaceZhe WangKee-Lung ChangZhen ChenKei FujiwaraZhenfang DuKei NakajimaZheng ChangKei ShinodaZheng Feei MaKeigo IkedaZhengxin CaiKeiichi IshidaZhibo GaiKeiichi MatsubaraZhichao FanKeiko HosohataZhi-Fu WuKeisaku SatoZhihao WuKeisuke HitachiZhihong YuanKeisuke SuzukiZhijun LiKeita KaiZhijun ZhouKeita WadaZhixiang WangKeith BonifaceZhonghua SunKeith CouperZisman DevyKelly A LofflerZlatko KopeckiKelly HoltZoe MoonKelly LambertZofia DrzazgaKelly LevanoZofia T. BilińskaKelly MorganZoltán János VerébKelsey E. UfholzZoubir DjeradaKelsey H. CollinsZsolt BaloghKelvin AfrashtehfarZsolt TothKelvin Ian AfrashtehfarZsuzsa Jenei-LanzlKen Farrington


